# In vitro and in vivo neuroprotective effects of cJun N-terminal kinase inhibitors on retinal ganglion cells

**DOI:** 10.1186/s13024-016-0093-4

**Published:** 2016-04-21

**Authors:** Byung-Jin Kim, Sean M. Silverman, Yang Liu, Robert J. Wordinger, Iok-Hou Pang, Abbot F. Clark

**Affiliations:** North Texas Eye Research Institute, University of North Texas Health Science Center, 3500 Camp Bowie Boulevard, Fort Worth, TX 76109 USA; Department of Pharmaceutical Sciences, University of North Texas Health Science Center, Fort Worth, TX 76107 USA; Department of Cell Biology & Immunology, University of North Texas Health Science Center, Fort Worth, TX 76107 USA; Present Address: Department of Ophthalmology, Johns Hopkins University School of Medicine, 400 N. Broadway, Baltimore, MD 21231 USA

**Keywords:** Retinal ischemia, c-Jun N-terminal kinase (JNK), Retinal ganglion cells, JNK inhibitors, Electroretinography

## Abstract

**Background:**

The c-Jun N-terminal kinase (JNK) signaling pathway plays an important role in neuronal pathophysiology. Using JNK inhibitors, we examined involvement of the JNK pathway in cultured rat retinal ganglion cell (RGC) death and in mouse retinal ischemia/reperfusion (I/R) injury of the visual axis. The in vitro effects of JNK inhibitors were evaluated in cultured adult rat retinal cells enriched in RGCs. Retinal I/R was induced in C57BL/6J mice through elevation of intraocular pressure to 120 mmHg for 60 min followed by reperfusion. SP600125 was administered intraperitoneally once daily for 28 days. Phosphorylation of JNK and c-Jun in the retina was examined by immunoblotting and immunohistochemistry. The thickness of retinal layers and cell numbers in the ganglion cell layer (GCL) were examined using H&E stained retinal cross sections and spectral domain optical coherence tomography (SD-OCT). Retinal function was measured by scotopic flash electroretinography (ERG). Volumetric measurement of the superior colliculus (SC) as well as VGLUT2 and PSD95 expression were studied.

**Results:**

JNK inhibitors SP600125 and TAT-JNK-III, dose-dependently and significantly (*p* < 0.05) protected against glutamate excitotoxicity and trophic factor withdrawal induced RGC death in culture. In the I/R model, phosphorylation of JNK (pJNK) in the retina was significantly (*p* < 0.05) increased after injury. I/R injury significantly (*p* < 0.05) decreased the thickness of retinal layers, including the whole retina, inner plexiform layer, and inner nuclear layer and cell numbers in the GCL. Administration of SP600125 for 28 days protected against all these degenerative morphological changes (*p* < 0.05). In addition, SP600125 significantly (*p* < 0.05) protected against I/R-induced reduction in scotopic ERG b-wave amplitude at 3, 7, 14, 21 and 28 days after injury. SP600125 also protected against the I/R-induced losses in volume and levels of synaptic markers in the SC. Moreover, the protective effects of SP600125 in the retina and SC were also detected even with only 7 days (Days 1–7 after I/R) of SP600125 treatment.

**Conclusions:**

Our results demonstrate the important role the JNK pathway plays in retinal degeneration in both in vitro and in vivo models and suggest that JNK inhibitors may be a useful therapeutic strategy for neuroprotection of RGCs in the retina.

**Electronic supplementary material:**

The online version of this article (doi:10.1186/s13024-016-0093-4) contains supplementary material, which is available to authorized users.

## Background

Activation of c-Jun N-terminal kinase (JNK) is frequently observed in degenerative diseases in mammalian neuronal system such as Alzheimer’s disease, Parkinson’s disease and glaucoma [1–3]. As a member of the mitogen activated protein (MAP) kinase family, activation of JNK is mediated by phosphorylation of various biological and environmental stress factors [4, 5]. In particular, several major inflammatory cascades, including oxidative stress, inflammatory cytokines, pattern-recognition receptors, and neurotransmitter receptors, activate the JNK signaling pathway [6–9] followed by activation of downstream signaling molecules such as c-Jun, ATF-2, ELK-1, and Stat3 [4, 5, 10]. These molecular events result in gene transcription associated with various biological outcomes including neuronal cell migration, cytoskeletal reorganization, and cell death [11–15].

As part of the central nervous system, the visual system, including retinal ganglion cells (RGC) and other neurons, has been investigated to identify the influence of JNK activation under various pathologic conditions. Tezel et al. demonstrated that JNK signaling is associated with RGC degeneration induced by tumor necrosis factor (TNF) receptor after optic nerve (ON) injury [16]. Bessero et al. reported that inhibition of JNK1 protected the retina from NMDA-induced excitotoxicity [17]. In addition, combined knockdown of *Jnk2* and *Jnk3* induced long-term protection of RGCs against axonal injury in mice [18]. Balaiya et al. also observed increased phosphorylated JNK (pJNK) in cultured RGCs exposed to hypoxic conditions [19]. More recently, Welsbie et al. showed that knockdown of the dual leucine zipper kinase, which is an upstream activator of JNK, improved survival and function of RGCs [20]. Taken together, the JNK pathway appears to play a pivotal role in RGC death under various insults and disease conditions.

Ischemia and subsequent reperfusion elicits severe damage in the visual system, leading to irreversible vision loss in many ocular diseases including retinal vessel occlusion, glaucoma, and diabetic retinopathy [21–23]. In particular, ischemia/reperfusion (I/R) injury in the retina causes RGC death, resulting in functional failure of transmitting visual information to specific receptive fields in the brain [24–26]. We previously reported that I/R damage in the retina induced morphological and functional degeneration and RGC death that was associated with temporal regulation of retinal gene expression [27]. In particular, various gene clusters, especially those related to cell death and inflammatory responses, were upregulated post injury and directly associated with the JNK signaling pathway in pathological stages of various diseases [28].

In this study, we evaluated the role JNK signaling pathway plays in retinal degeneration and RGC death using pharmacological JNK inhibitors in retinal cell culture and mouse retinal I/R injury models. We first examined their protective effects against cell death in an adult rat retinal cell culture. We further examined the effect of JNK inhibition on I/R-induced changes in the retina and SC. We found that JNK inhibition provided total morphological and functional protection to RGCs.

## Results

### Protection of RGC death by JNK inhibitors

**Fig. 1:**
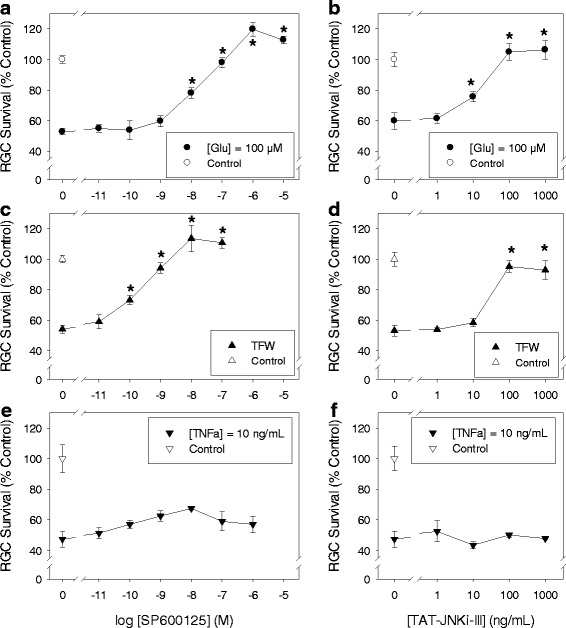
JNK inhibitors protected against RGC death induced by glutamate (Glu) excitotoxicity and trophic factor withdrawal (TFW), but not by tumor necrosis factor (TNF)-α*.* Cultured adult rat retinal cells were treated with the indicated concentration of JNK inhibitors SP600125 (**a**, **c**, **e**) or TAT-JNKi-III (**b**, **d**, **f**) in the presence of the cytotoxic insults: 100 μM of Glu (**a**, **b**), TFW (**c**, **d**), or 10 ng/mL TNFα (**e**, **f**) for 3 days. Surviving cells were fixed and labeled with anti-Thy-1 antibody and manually counted. Vehicle-treated (no insult) control group in each study defines 100 %. Symbols represent mean ± SEM (*n* = 6–10). Asterisk indicates statistical difference (*p* < 0.05) between the JNK inhibitor-treated groups versus the respective insult-only group by One-way ANOVA then Dunnett’s test

Several insults are known to induce cell death of purified RGCs in vitro. Otori et al. showed that glutamate (5 to 500 μM) induced cell death of cultured rat RGCs in a dose-dependent manner [29]. Withdrawal of trophic factors also induced cultured RGC death [30]. In addition, TNFα from glia under ischemic conditions also induced RGC death in a co-culture system [31]. Based on previous findings, we further investigated whether these RGC death mechanisms are associated with JNK signaling. Death of cultured RGCs was induced by treating cells for 3 days with glutamate (100 μM), TNFα (10 ng/mL), or TFW (trophic factor withdrawal) in the presence or absence of various concentrations of the JNK inhibitors SP600125 or TAT-JNKi-III. Cells were then fixed and labeled with anti-Thy-1 antibody for RGC counting. SP600125 treatment significantly (*p* < 0.05) enhanced RGC survival in a dose-dependent manner against glutamate and TFW-induced cell death (Fig. 1a and c), but did not protect against TNFα-induced RGC death (Fig. 1e). Similarly, TAT-JNKi-III also dose-dependently protected against RGC death induced by glutamate and TFW, but not by TNFα (Fig. 1b, d and f).

### JNK activation induced by retinal I/R

**Fig. 2:**
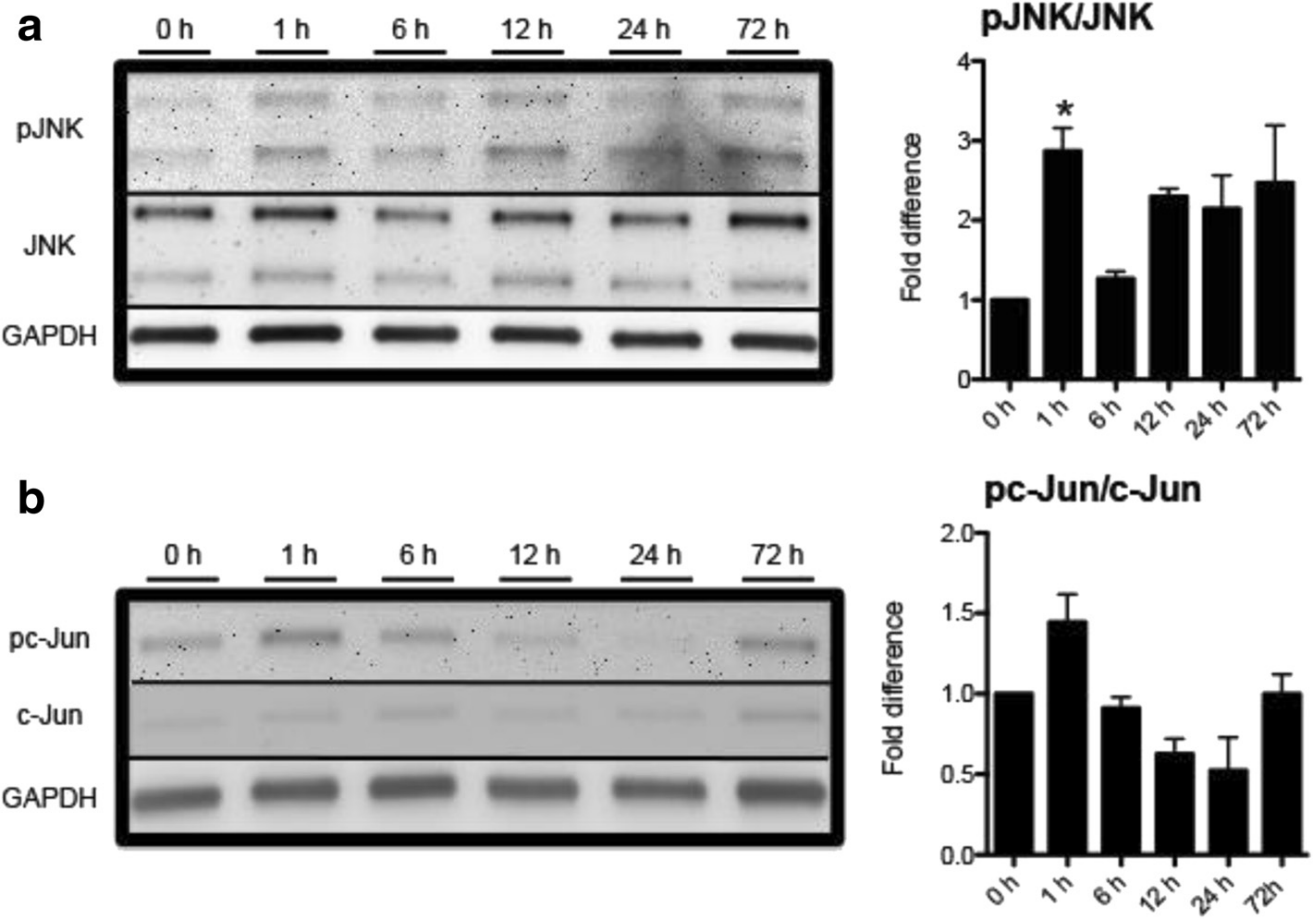
Retinal I/R induced phosphorylation of JNK and c-Jun*.* Mouse retinas were collected at 1, 6, 12, 24, and 72 h post I/R injury. The 0 h control represents the non-injured group. Western blotting analyses were conducted using total retinal proteins. **a** Representative images of phosphorylated JNK (pJNK), total JNK, and loading control GAPDH as well as ratio of pJNK versus total JNK, analyzed by ImageJ. JNK phosphorylation was significantly (*p* < 0.05) increased at 1 h after I/R injury by 2.87 (±1.87) fold compared to 0 h control. **b** Representative images of phosphorylated c-Jun (pc-Jun), total c-Jun, and loading control GAPDH as well as ratio of pc-Jun versus total c-Jun analyzed by ImageJ. Bars represent mean ± SEM (*n* = 3). *Asterisk* indicates statistical difference (*p* < 0.05) compared to the 0 h control group by One-way ANOVA then Dunnett’s test

JNK is activated via phosphorylation of threonine and tyrosine residues located in the activation loop in the carboxyl-terminus. Activated JNK subsequently phosphorylates c-Jun [32, 33]. Therefore, we examined I/R-induced phosphorylation of JNK and c-Jun in the whole retina at various time points after injury using immunoblotting analysis (Fig. 2). Retinal JNK phosphorylation was detected at 0, 1, 6, 12, 24, and 72 h after I/R injury. As previously reported, we also observed a basal level of phosphorylated JNK at 0 h [34, 35]. JNK phosphorylation appeared to show a bi-phasic increase with an initial peak at 1 h (*p* < 0.05 vs. 0 h control) and a delayed increase at 72 h after I/R injury (Fig. 2a). Interestingly, c-Jun phosphorylation also closely correlated with the JNK phosphorylation pattern (Fig. 2b).

**Fig. 3:**
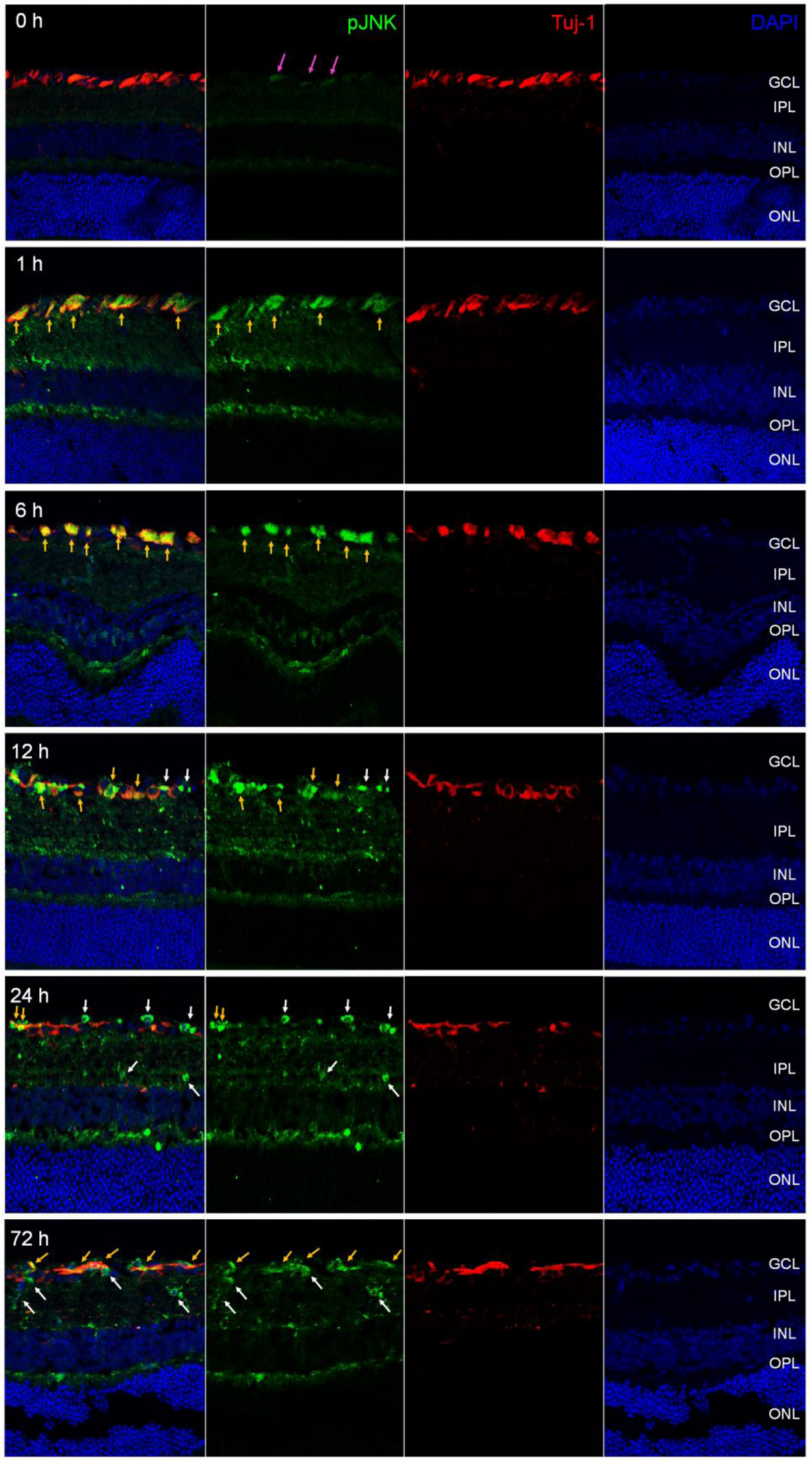
Phosphorylated JNK was detected in retina after I/R injury*.* Frozen-sectioned (10 μm) retina samples from 0, 1, 6, 12, 24 and 72 h after I/R injury were used for immunohistochemistry. Phosphorylated JNK was detected (*green fluorescence*). Tuj-1 immunofluorescence (*red*) was used as RGC marker. DAPI staining (*blue*) represents cell nuclei for counter staining. The *magenta arrows* represent basal JNK phosphorylation in 0 h retina. All *yellow arrows* represent phosphorylated JNK in RGC and *white arrows* represent non-RGC in GCL

**Fig. 4:**
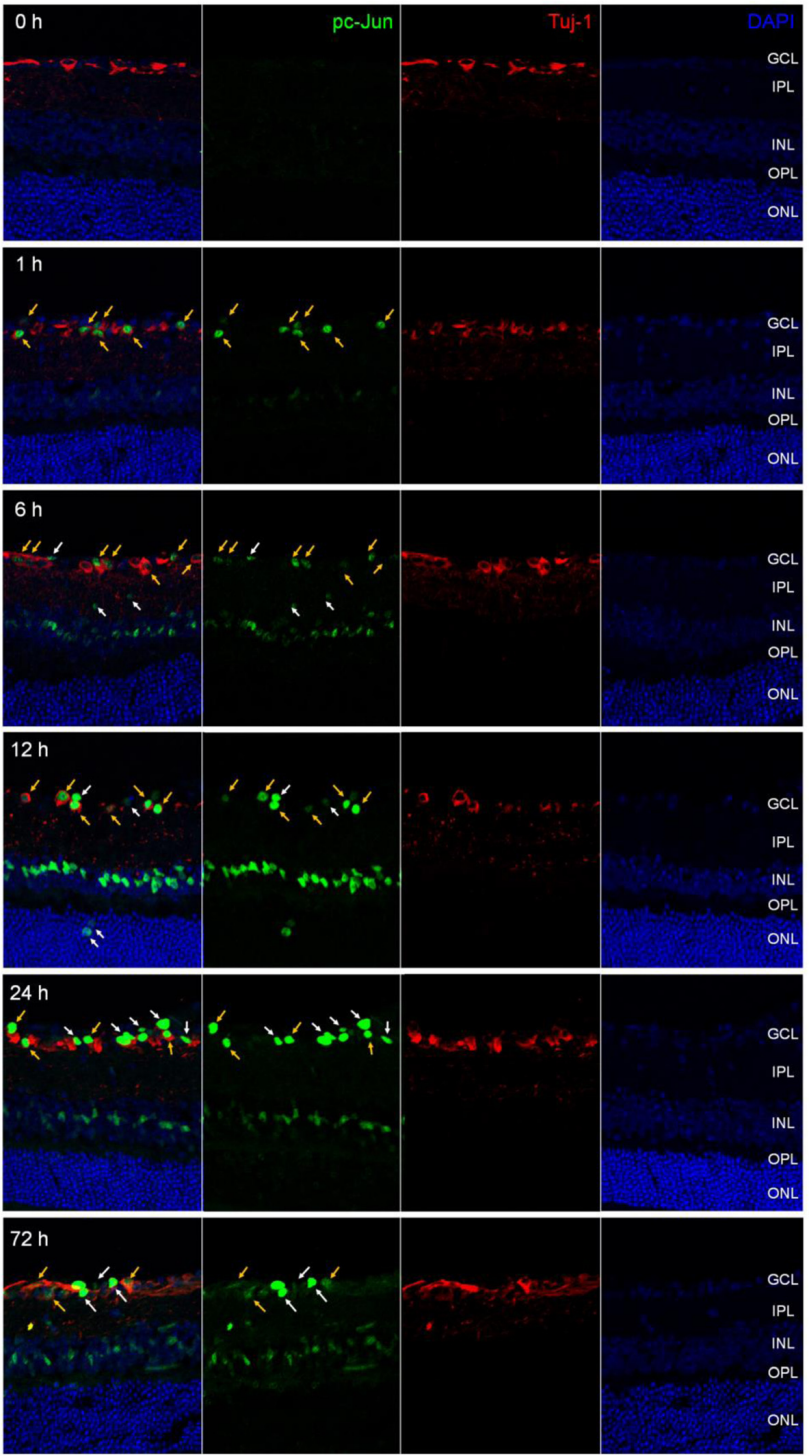
Phosphorylated c-Jun was detected in retina after I/R injury*.* Frozen-sectioned (10 μm) retina samples from 0, 1, 6, 12, 24 and 72 h after I/R injury were used for immunohistochemistry. As with JNK, phosphorylated c-Jun was detected (*green fluorescence*). Tuj-1 immunofluorescence (*red*) was used as RGC marker. DAPI staining (*blue*) represents cell nuclei for counter staining. All *yellow arrows* represent phosphorylated c-Jun in RGC and *white arrows* represent non-RGC in GCL

**Fig. 5:**
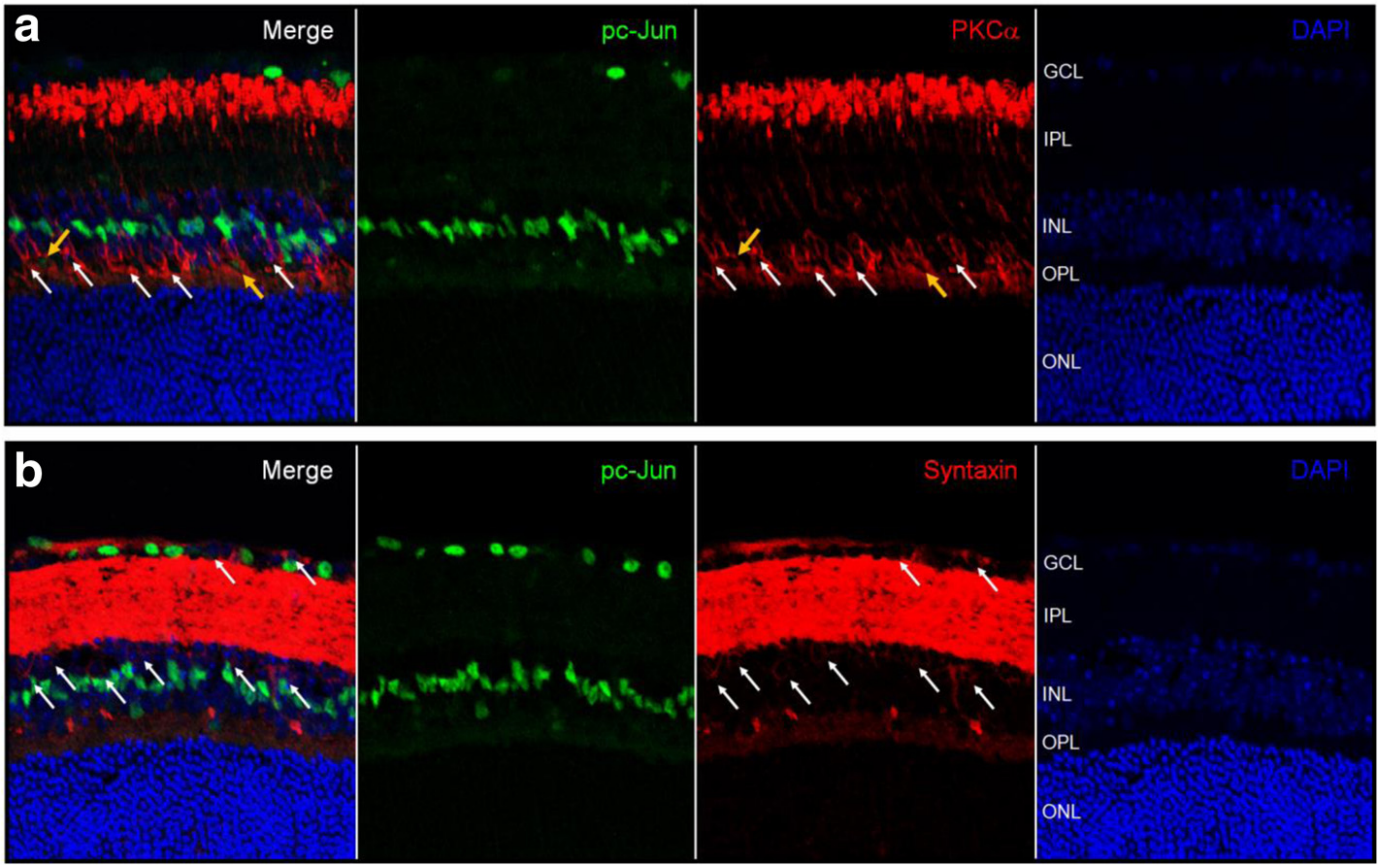
Phosphorylated c-Jun was not co-localized with both bipolar cell and amacrine cells in retina after I/R injury*.* 10 μm frozen retinal sections from 0, 1, 6, 12, 24 and 72 h after I/R injury were used to detect phosphorylated c-Jun (*green fluorescence*) in bipolar cells (**a**) and amacrine cells (**b**). PKCα (**a**) or syntaxin (**b**) immunofluorescence (*red*) were used to identify bipolar cells or amacrine cells in INL. DAPI staining (*blue*) represents cell nuclei for counter staining. All phosphorylated c-Jun in INL were indicated using *white arrow* (no co-localization) or *yellow arrow* (co-localization) to show its location in INL with amacrine cells (**a**) and bipolar cells (**b**)

In immunohistochemical analysis, basal level of JNK phosphorylation was observed in the same location with the RGC marker Tuj-1(magenta arrows) and OPL corresponding with our immunoblotting results. I/R injury induced drastic increase of JNK phosphorylation in Tuj-1 positive RGCs at early post-I/R injury times (1 h and 6 h) and detected in non-RGCs (white arrows) from 12 h to 72 h after I/R injury (Fig. 3). Notably, JNK phosphorylation was also observed in the INL from 6 h to 12 h after I/R injury, and in the IPL from 24 h to 72 h. In addition, strong JNK phosphorylation was continuously observed in OPL from 1 h to 72 h after I/R injury (Fig. 3). Phosphorylated c-Jun was also detected in a very similar manner to pJNK in I/R-injured retinas. Phosphorylated c-Jun was first observed in Tuj-1-positive RGCs at 1 h (yellow arrows) after I/R injury, and the presence of pc-Jun was continuously detected in both RGCs and non-RGCs (white arrow) from 6 h to 72 h in GCL after I/R injury (Fig. 4). As we also observed phosphorylation of pc-Jun in INL, we further stained phosphorylated c-Jun with bipolar cell (PKCα) and amacrine cell (syntaxin) markers at 24 h after I/R injury. Interestingly, phosphorylated JNK and c-Jun was not co-localized with either bipolar or amacrine cells (white arrow) (Fig. 5).

### Protection against I/R-induced retinal damage by SP600125

**Fig. 6:**
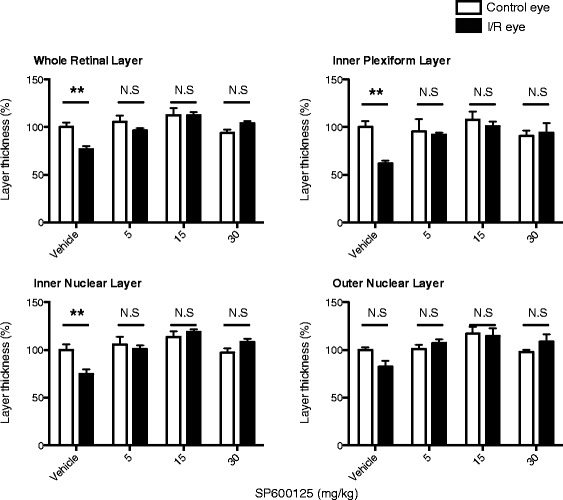
SP600125 protected against I/R-induced thinning of inner retinal layers*.* Three doses of SP600125 (5, 15, and 30 mg/kg) or vehicle were administered intraperitoneally 2 h prior to retinal I/R injury followed by once daily dosing. After 28 days, histological changes in retinal thickness, including whole retina (from NFL to ONL), IPL, INL and ONL were analyzed. Data represent mean ± SEM (*n* = 5) at each time point. The mean thickness of the uninjured, vehicle-treated retinas was set as 100 %. In vehicle-treated group, I/R injury significantly reduced whole retina thickness 23.2 ± 5.7 % compared to non-injured contralateral retinas. In particular, IPL was reduced 38.0 ± 6.7 % and INL reduced 25.1 ± 7.4 %. No significant layer change was observed in ONL. In contrast, no significant layer thickness reduction was observed from SP60025-administered groups at each dose (5, 15 and 30 mg/kg). **: *p* < 0.01; NS: non-significant between contralateral control and I/R eyes by two-tailed, paired *t*-test

**Fig. 7:**
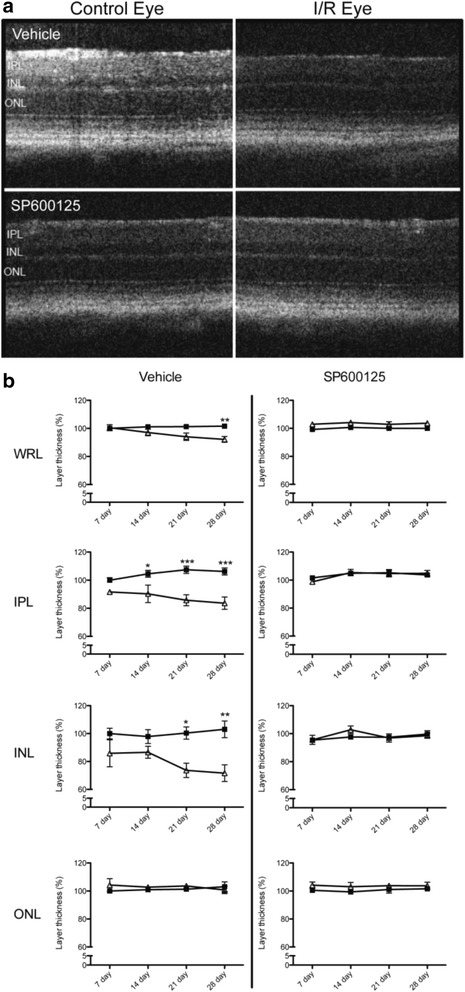
SP600125 ameliorated I/R-induced progressive retina thinning*.* Retinal I/R injured mice were treated with SP600125 (15 mg/kg) or vehicle for 28 days. SD-OCT was used to determine progressive changes in whole retinal layer (from NFL to ONL), and each of IPL, INL, and ONL. **a** Representative images showing layer changes after 28 days post I/R injury. **b** Quantitative analyses of progressive changes of thickness of each layer at different time points. Open symbols: I/R eyes; closed symbols: un-injured contralateral eyes. In vehicle-administered groups, whole retina thickness in I/R-injured eyes were progressively decreased at 28 days of injury compared to contralateral non-injured eyes. These changes were mainly observed in inner retinal layers, IPL and INL. In contrast, SP600125 administration inhibited I/R-induced layer reduction. Data are shown as mean and SEM (*n* = 4). Two-way ANOVA analyses indicate that there were no significant time-dependent changes in all SP600125 groups, but significant differences were detected between the control and I/R groups at certain time points (*: *p* < 0.05, **: *p* < 0.01, ***: *p* < 0.001 by Bonferroni’s post-hoc test). (WRL: whole retina layer, NFL: nerve fiber layer, IPL: inner plexiform layer, INL: inner nuclear layer, ONL: outer nuclear layer)

**Fig. 8:**
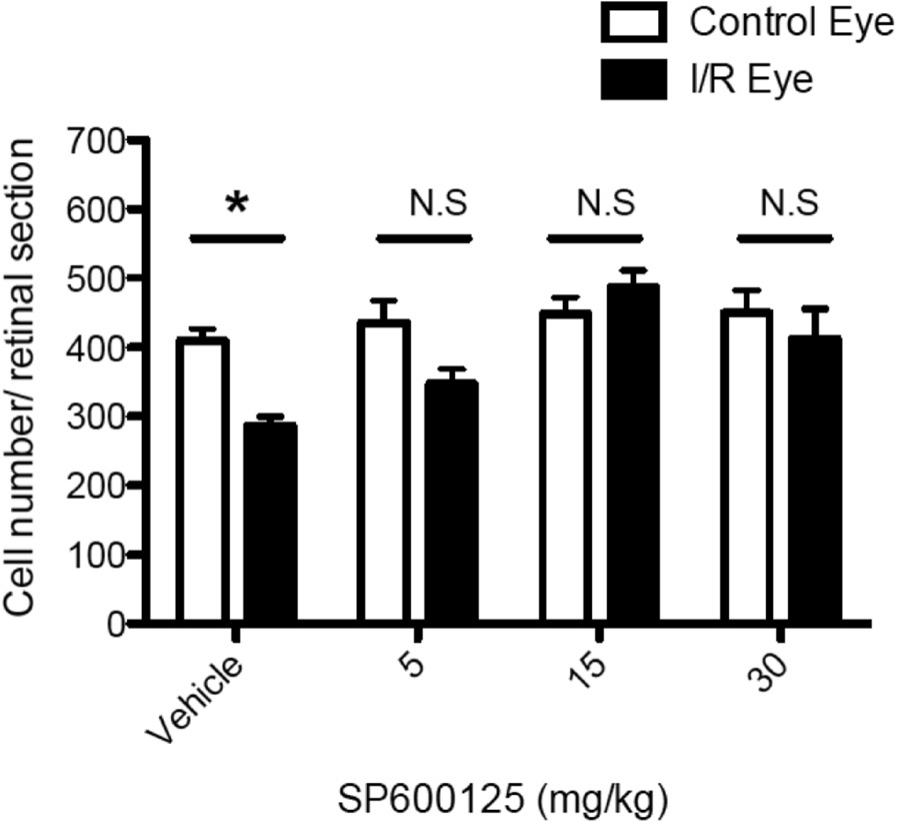
SP600125 protected against I/R-induced cell loss in the GCL. Total cell numbers in the GCL were counted from retinas of vehicle or SP600125-treated animals at 28 days after I/R injury. Non-injured contralateral eyes were also analyzed for comparison. I/R injury induced significant GCL cell loss (30.0 ± 5.6 %) compared to contralateral non-injured eyes whereas SP600125 administration showed total protection. Data represent mean ± SEM (*n* = 5). *: *p* < 0.05; NS: non-significant between contralateral control and I/R eyes by two-tailed, paired Student’s *t*-test

**Fig. 9:**
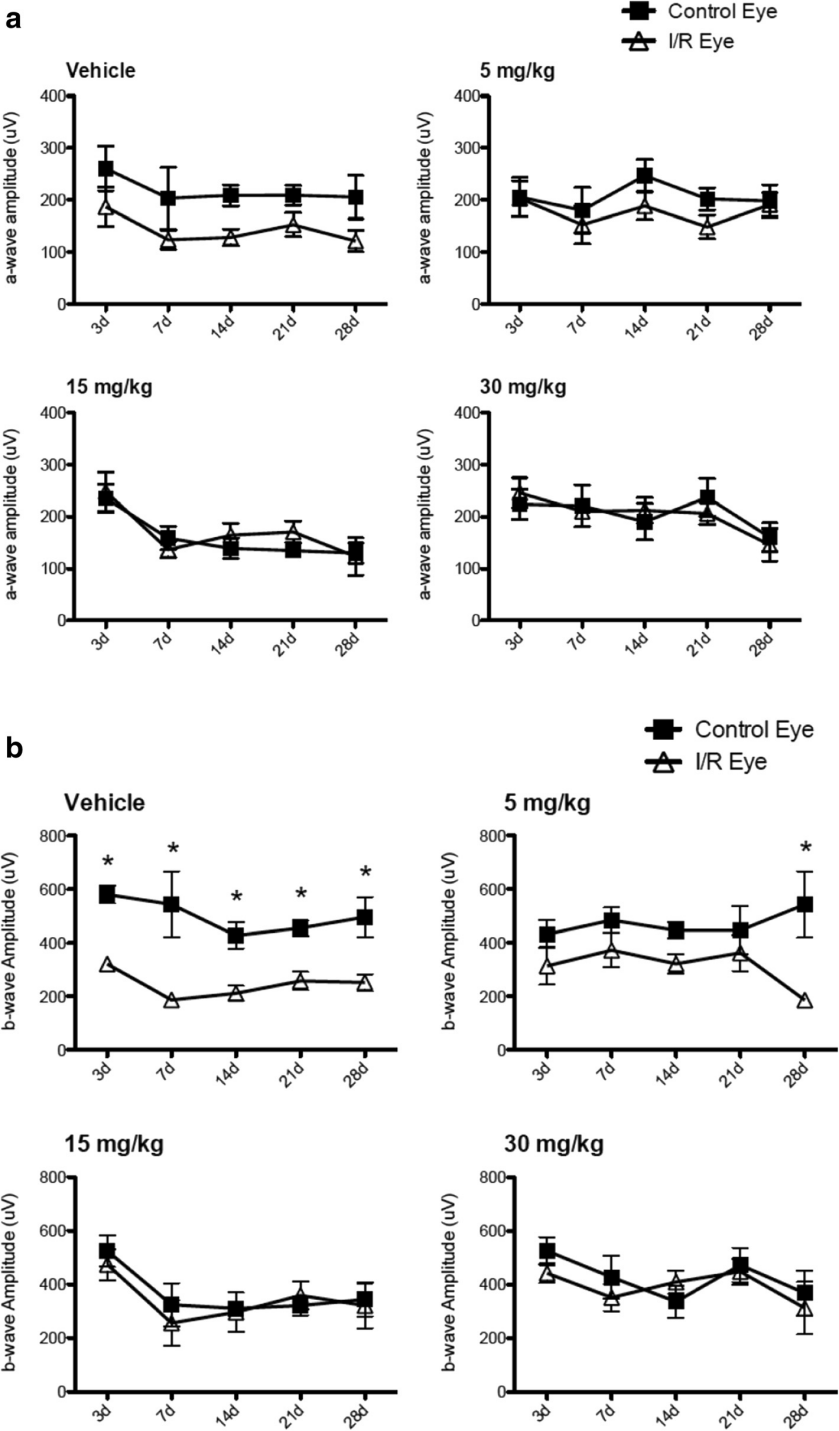
SP600125 ameliorated the I/R-induced reductions in ERG b-wave amplitudes*.* The a- and b- wave amplitudes of scotopic ERG at 10 cd light intensity were measured in the vehicle and SP600125-treated groups 3, 7, 14, 21, and 28 days after I/R. Data represent mean ± SEM (*n* = 5). I/R injury induced slight reductions of a-wave amplitudes (**a**) and significant (*p* < 0.05) impairment of b waves (**b**) over 28 days in the vehicle group. In contrast, daily administration of SP600125 prevented impairment of a-waves and b-waves (**a** and **b**). Two-way ANOVA analyses indicate that there were no significant time-dependent changes in all SP600125 groups, but significant differences were detected between the control and I/R groups at certain time points (*: *p* < 0.05 by Bonferroni’s post-hoc test)

Previously, it was shown that systemic administration of SP600125 showed protection in neurodegenerative animal models such as focal brain ischemia, in which the JNK signaling pathway are involved [36]. Therefore, we administered SP600125 to mice in our model of retinal I/R to determine potential protective effects of JNK inhibition based on our in vitro data. We preliminarily verified in vivo inhibition of SP600125 against JNK and c-Jun phosphorylation after retinal I/R injury. SP600125 (30 mg/kg) was administered intraperitoneally 2 h prior to I/R injury followed by immunostaining analysis for phosphorylated JNK and c-Jun after 24 h. As expected, SP600125 administration decreased pc-Jun phosphorylation in both the GCL and INL (Additional file 1: Figure S9). Three different doses of SP600125 (5, 15, 30 mg/kg) or vehicle were administered intraperitoneally as described above followed by once daily administration for 28 days after I/R injury. Histological measurement of H&E stained retinas showed that I/R in the vehicle control group caused significant (*p* < 0.01) reduction of retinal layer thickness, particularly in the IPL and INL over 28 days (Fig. 6) as previously reported [27]. Importantly, administration of SP600125 at each of the three doses completely prevented I/R-induced thinning of the IPL and INL (Fig. 6). In addition to histology, we also examined morphological changes of retina using SD-OCT at various time points after I/R injury, with or without SP600125 (15 mg/kg) treatment. I/R injury caused progressive and significant (*p* < 0.05) thinning of the IPL and INL, whereas SP600125 treatment ameliorated these changes (Fig. 7). I/R-induced cell loss in the GCL was also significantly attenuated by all three doses of SP600125 (Fig. 8). In addition, I/R injury significantly (*p* < 0.05) reduced the scotopic ERG b-wave (Fig. 9b). The I/R-mediated reduction in b-wave amplitudes were also restored by SP600125 treatment (Fig. 9b).

### Protection against I/R-induced degeneration of RGC axons in the SC by SP600125

**Fig. 10:**
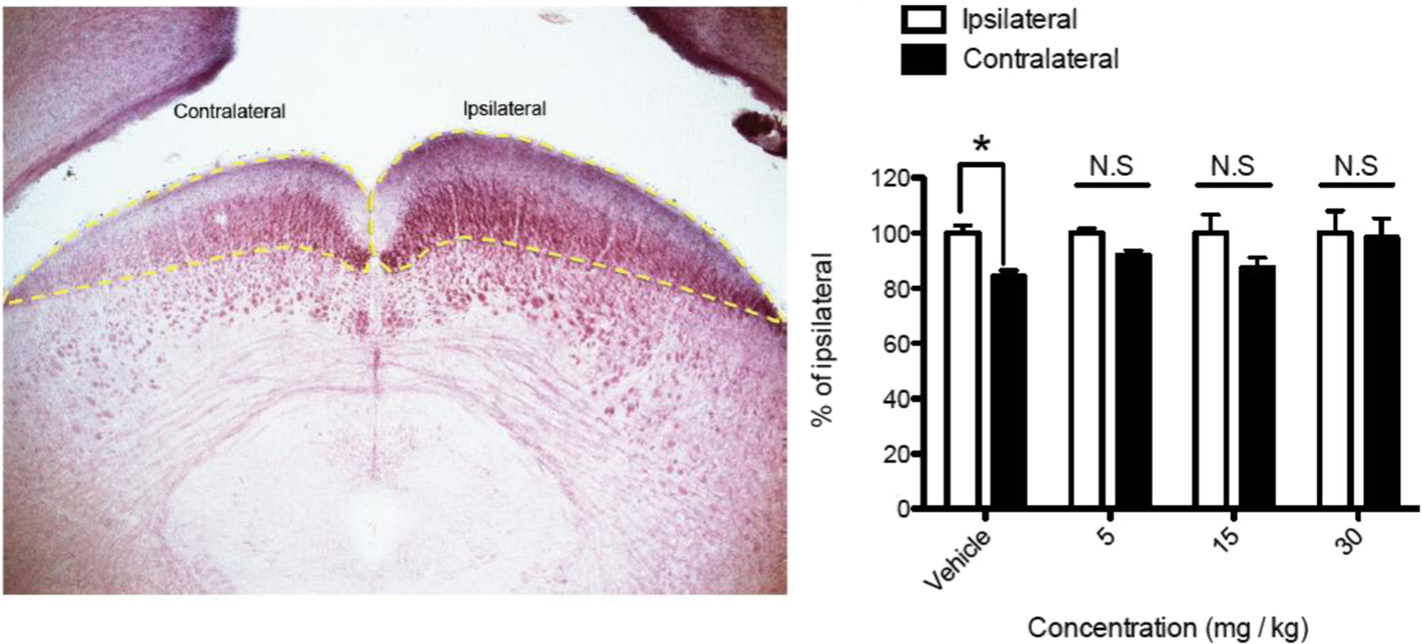
SP600125 protected retinal I/R-induced reduction in size of superior colliculus*.* Regions of mouse brains containing SC from vehicle or SP600125 (5, 15, 30 mg/kg)- treated groups after 28 days post retinal I/R injury were collected for serial frozen sectioning and black gold staining. Bright field images were taken and cross-sectional area of superficial layers from each contralateral or ipsilateral side were measured and averaged in series of all stained sections. In vehicle treated groups, I/R injury induced significant area volume loss (~16 %) in the contralateral SC region compared to the ipsilateral region. However, SP600125 administration protected SC from I/R-induced area volume loss in contralateral hemispheres. Data represent mean ± SEM (*n* = 4–5). The ipsilalateral area volume in each brain was set as a 100 %. *: *p* < 0.05 between contralateral and ipsilateral volumes of SC by two-tailed, paired *t*-test

**Fig. 11:**
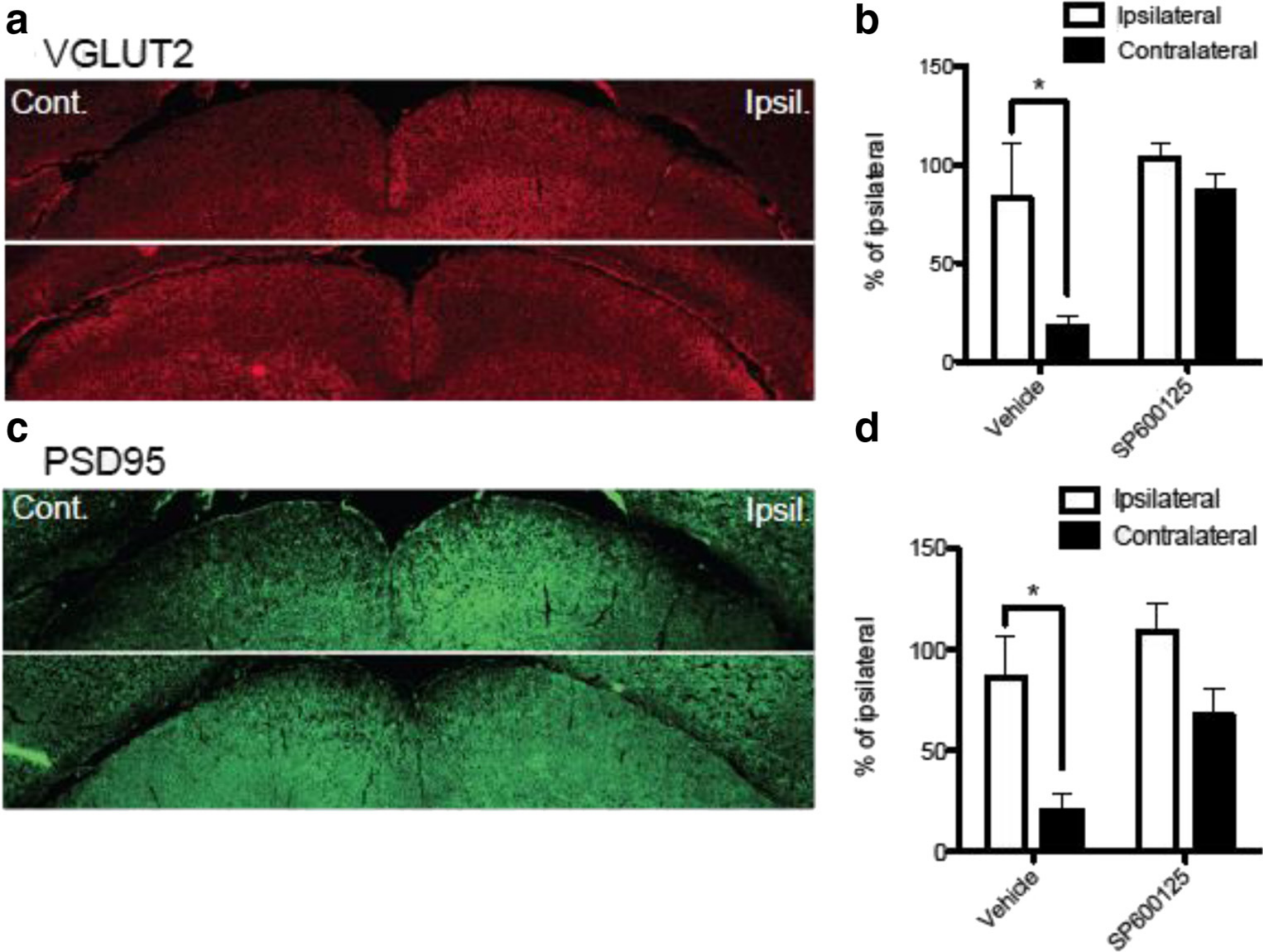
SP600125 prevented I/R-induced impaired expression of synaptic markers VGLUT2 and PSD95 in the superior colliculus*.* Mouse brains from vehicle or SP600125 (15 mg/kg) treatment groups were collected for immunohistochemistry at 28 days post retinal I/R injury. Each brain section was incubated with primary antibody for presynaptic marker VGLUT2 or post-synaptic marker PSD95. **a** and **c** Images including both ipsilateral (Ipsil) and contralateral (Cont) superficial layers of the superior colliculus from vehicle treated (above) and SP600125 treated (below) mice. **b** and **d** Data represent mean ± SEM (*n* = 4 - 5). I/R injury resulted in significant fluorescence intensity loss of VGLUT2 (84.4 ± 19.0 %) and PSD95 (76.8 ± 12.6 %) in the contralateral SC after 28 days in vehicle-administered group (**b** and **d**). In contrast, 28-day administration of SP600125 prevented the loss of both synaptic markers (**b** and **d**). Total area volume of the ipsilateral SC defines 100 %. *: *p* < 0.05 between contralateral and ipsilateral volumes by two-tailed, paired Student’s *t*-test

In the mouse visual system, approximately 95 % of RGC axons project contralaterally [37], and at least 70 % of RGCs project to the superficial layer of SC [38]. As shown in Fig. 10, I/R injury caused a significant (*p* < 0.05) decrease (~20 %) in the superficial SC layer cross-sectional area in the contralateral side of the I/R injury compared to the ipsilateral side after 28 days. Importantly, administration of SP600125 (5, 15, 30 mg/kg) protected against this I/R-induced SC neuron decrease. We also assessed the synaptic terminal of RGC neurons in the SC superficial layer by examining expression of the presynaptic marker VGLUT2 (vesicular glutamate transporter 2). Furthermore, we also quantified intensity of the postsynaptic marker PSD95 to measure potential failure in post-synapses of RGC neurons in the SC. I/R injury significantly (*p* < 0.05) decreased expression of both VGLUT2 and PSD95 in the contralateral SC in comparison to the ipsilateral side after 28 days. Decreased expression of these two markers were attenuated by daily SP600125 administration for 28 days (Fig. 11).

### Protective effects in the retina and SC by only 7-day treatment with SP600125

**Fig. 12:**
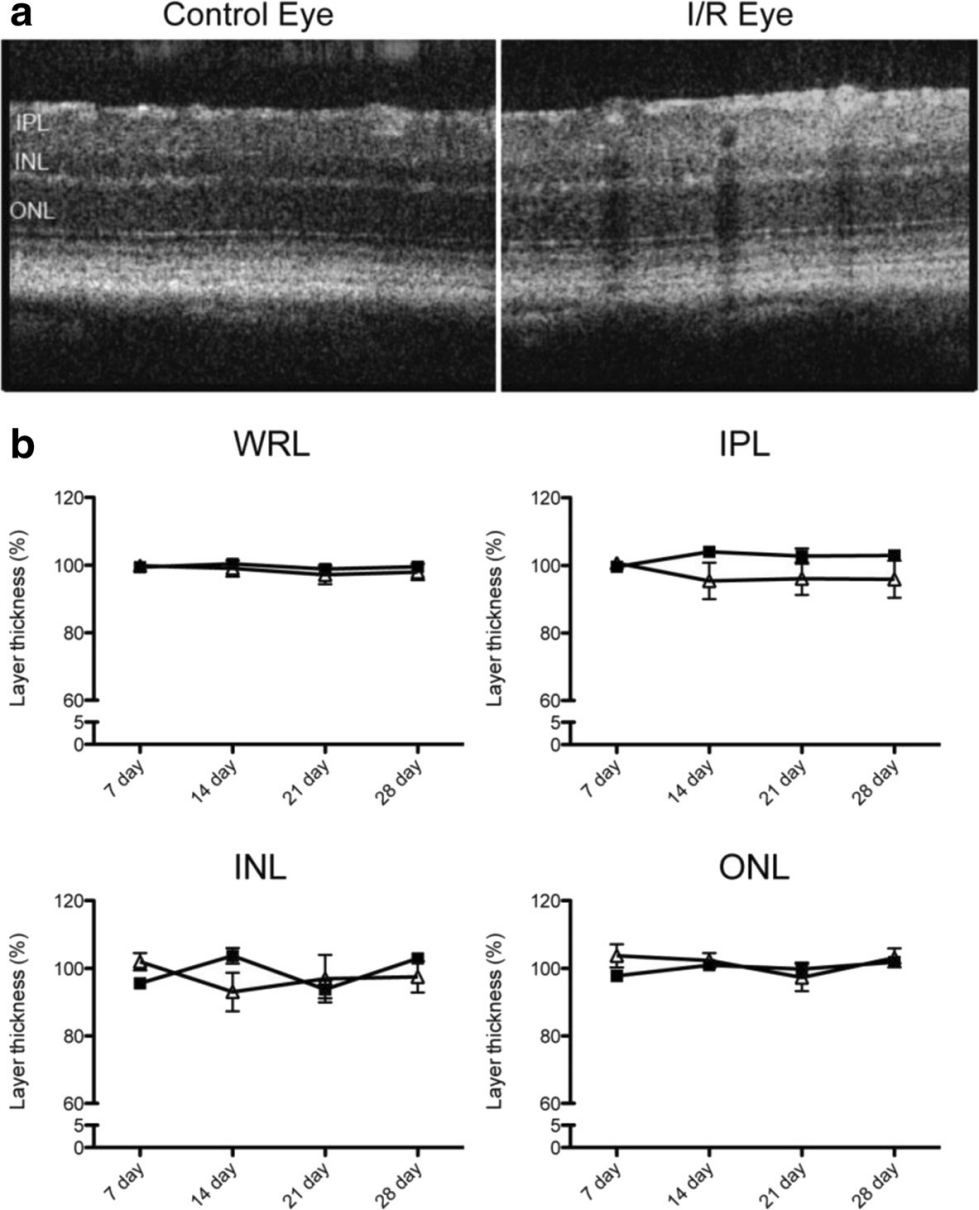
Seven-day administration of SP600125 also protected the retina from I/R-induced layer degeneration. SP600125 (15 mg/kg) was administered to retinal I/R injured mice for the first 7 days only. SD-OCT scanning was used to determine progressive changes in whole retinal layer (from NFL to ONL) or each sublayer including IPL, INL and ONL. Images were selected to show layer changes 28 days post I/R injury (**a**). Progressive changes of whole retinal layer or sub-layers were monitored (**b**). 7-day administration of SP600125 resulted in protection from retinal layer degeneration induced by I/R injury after 28 day. Open symbols: I/R eyes; closed symbols: un-injured contralateral eyes. Two-way ANOVA analyses indicate that there were no significant time-dependent or injury-dependent changes. (WRL: whole retina layer, IPL: inner plexiform layer, INL: inner nuclear layer, ONL: outer nuclear layer)

**Fig. 13:**
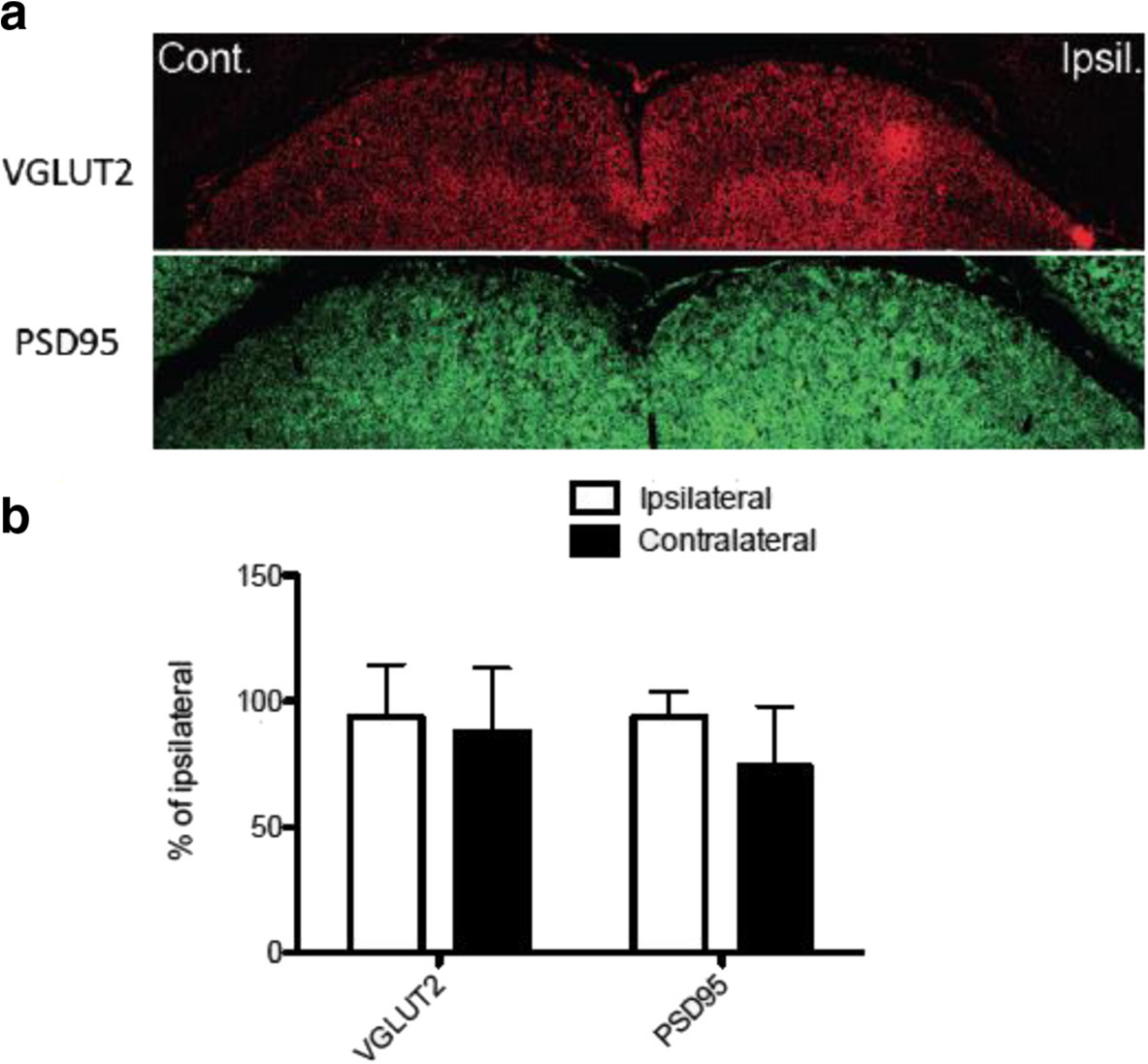
The 7-day administration of SP600125 protected I/R-induced reduction in VGLUT2 and PSD95 expression in the superior colliculus*.* (**a**) Mouse brains from 7-day SP600125 treatment (15 mg/kg) were collected for immunohistochemistry 28 days post retinal I/R injury. (**b**) Data represent mean ± SEM (*n* = 4) of percentages at each hemisphere from both contralateral (dark bars) and ipsilateral (open bars) sides compared to vehicle ipsilalateral volume, set as a 100 %. No significant difference was detected between contralateral and ipsilateral samples by two-tailed, paired Student’s *t*-test

The above results demonstrated that daily treatment with SP600125 for 28 days was protective against retinal I/R-induced damage to the retina and SC. To assess whether a shorter treatment period was also protective, SP600125 (15 mg/kg) was administered daily for only the first 7 days after I/R injury, and changes of the retina and SC were monitored for 28 days. I/R-induced degeneration of the INL and IPL was completely ameliorated by the 7-day administration of SP600125 as measured by SD-OCT (Fig. 12). Similarly, 7-day treatment with SP600125 also prevented the loss of synapse markers VGLUT2 and PSD95 in the SC, showing protection from RGC axon loss (Fig. 13).

## Discussion

In this report, we demonstrated that JNK inhibitors were effective in protecting cultured adult rat RGCs from cytotoxicity, and also against neurodegeneration in the mouse of retinal I/R. In cultured adult rat retinal neurons, we confirmed previous findings that excitotoxicity, TFW, and TNFα result in loss of RGCs [30, 39]. We further showed that two selective JNK inhibitors, SP600125 and TAT-JNKi-III, protected RGCs in a dose-dependent manner against glutamate- and TFW-induced cell death, but not TNFα cytotoxicity. These findings agree with reports indicating the involvement of JNK following NMDA excitotoxicity in the rat retina [40, 41]. Furthermore, we established SP600125 as a neuroprotective agent to neurons in the mouse retina and SC following retinal I/R. To our knowledge, this is the first study to validate the involvement of the JNK pathway both in cytotoxic injury to cultured primary retinal neurons, as well as in vivo following retinal I/R, through multiple functional and morphological assessments of these neural tissues.

We previously reported retinal I/R injury caused progressive morphological and functional degeneration in the retina. We observed strong temporal correlation with various pathological markers, including those related to proinflammatory response and cell death [27]. Here, we extend those characterizations and further identify significant increases in phosphorylation of JNK and its downstream target c-Jun. Strong activation of JNK and c-Jun signaling has been reported in animal models of CNS ischemia. In transient forebrain ischemia induced by occlusion of the common carotid artery, there were significant increases in pJNK in hippocampal CA1 and CA3 neurons within 15 min of reperfusion [42–44]. Transient global ischemia induced by cardiac arrest in mice and rats similarly led to increased levels of active JNK in CA1 neurons [45, 46]. In a murine model of middle cerebral artery occlusion, pJNK was increased in the cerebral cortex and striatum [47–49]. Finally, in similar models of retinal I/R, active JNK signaling has been identified throughout the neural layers of the retina 24 h post ischemia [50–52].

Activation of JNK occurs in many neurodegenerative diseases in addition to ischemic damage. Cerebral cortex and hippocampal sections from Alzheimer’s disease patients have markedly increased pJNK staining co-localized with neurofibrillary tangles [53]. In tissue samples from Parkinson’s disease (PD) patients, pJNK staining was in close proximity to Lewy bodies. Likewise, in the MPTP mouse model of PD, there was significantly increased activation of the JNK pathway; and dual *Jnk2/3* knockout mice were protected against the neurodegenerative effects of this toxin [2, 54]. Protective strategies aimed at the reduction or removal of JNK through the use of mouse genetics, viral vectors, and small molecule inhibitors support the involvement of JNK signaling in multiple neurodegenerative conditions.

The biphasic increase in pJNK and pc-Jun in our study occurred over a 72 h time period. While our report nicely showed the sustained expression of pJNK several hours to days following reperfusion, it is interesting that the most significant increases of pJNK and pc-Jun occurred 1 h following reperfusion, which were observed mainly in the Tuj-1 positive RGC cell population. Among the earliest responses to reperfusion of occluded blood flow are influx of ions, excitatory neurotransmitter release, and free radical formation [23]. Reactive oxygen species (ROS) such as superoxide are potent activators of JNK signaling, acting far upstream on the MAP3K ASK1. This kinase-mediated activation by ROS facilitates apoptosis in the mitochondria, as JNKs are known to activate caspases and induce cytochrome c release [55, 56]. Thus, our observed early activation of JNK signaling may be attributed to the increase in free-radical production resulting from reperfusion, and RGC may be the most susceptible cell type to trigger JNK signaling by this microenvironmental stress. Previously, Roth and colleagues showed increased pJNK within 6 h after I/R [57]. This apparent discrepancy in temporal retinal JNK activation may be explained by slight differences between the models. Ross et al. used a milder elevation of IOP to induce retinal ischemia in rats, while our study was done in mice. Nonetheless, both studies indicate that JNK phosphorylation is widespread in many cell types in the inner retina, including the GCL, IPL and INL. In support, Schmid et al. showed that I/R injury induced loss of amacrine cells, but not rod bipolar cells in INL [58]. Together, these studies indicate that I/R injury may trigger the activation of JNK pathway in association with diverse microenvironmental changes in inner retina and promote the cellular degeneration based on the different susceptibility of each cells to specific initiating factors.

Notably, we also observed that phosphorylated c-Jun was not co-localized with amacrine cells and bipolar cells, although we observed strong c-Jun phosphorylation in INL from 6 h to 72 h after I/R injury (Fig. 5). In contrast, Gesslein et al. showed increased phosphorylated c-Jun signals in Müller cell bodies in the INL after I/R injury [59]. In retinal I/R injury, Müller cell gliosis is a major marker associated with their cellular changes causing pathological progression [27]. Importantly, several in vitro studies suggested that JNK pathway may be involved in Müller cell activation related to proinflammatory cytokine production and modified glutamate-uptake, which are also important I/R-induced molecular stressors to determine the fate of retinal pathology [60, 61]. Although further characterization is necessary, our observations suggest that the cells co-localized with phosphorylated c-Jun may be Müller cells based on their locational polarity in the INL toward vitreous side [62]. In addition, our data also suggest the diversity in the role of the JNK pathway in different retinal cell types that may result in different cellular fates such as programmed cell death or inflammatory activation.

A number of strategies have been developed for inactivation of the JNK pathway, either directly targeting one or all of the JNK isoforms [18], the adapter protein JIP1 [63], or an upstream kinase such as MLK1/2/3 [64]. Inhibition of JNK activation has been previously shown to be an effective therapeutic strategy in vitro and in vivo. Reduction of pJNK protected primary neuronal cultures from TFW [65]. In animal studies, JNK inhibition produced a marked reduction in lesion size resulting from brain ischemia and preserved neuronal health of affected tissues, decreased the number TUNEL-positive cells following excitotoxicity, and rescued against retinal thinning and cellular loss of RGCs [16–19, 41, 45, 65–67]. However, only a few studies have determined whether JNK signaling is the critical pro-apoptotic mediator of pathological structural and functional changes following retinal I/R injury.

SP600125 is a widely used inhibitor for all three JNK isoforms [68]. This compound showed significant protection in animal models of neurodegenerative disease [54], cerebral ischemia [69, 70], and retinal excitotoxicity [41]. We evaluated the neuroprotective effect of SP600125 in our model of retinal I/R injury. SP600125 protected against thinning of the inner neural retinal layers and cellular loss in the GCL. We used SD-OCT to temporally and non-invasively follow morphological changes in the retina of each mouse. Furthermore, we believe our study to be the first to also show complete preservation of retinal b-wave amplitude over a 28-day period after I/R injury. Our findings of neuroprotection are in disagreement with those previously published by Roth and colleagues, who reported a lack of protection by two different JNK inhibitors including SP600125 [57]. This discrepancy may be explained by the differences in dosing strategies. Our study effectively protected the retina through two different systemic dosing regimens that each required daily administration following reperfusion in addition to a dose given right before I/R injury. In contrast, Roth et al. used a single post-ischemic intravitreal dose of SP600125. A recent study showed JNK modulation and neuroprotection against retinal ischemia using crocetin, a carotenoid derivative [52]. However, their assessments were made only at 7 days post injury, and the long-term effectiveness was not reported.

Pathological changes related to inner retinal injury are not limited to just the retina because RGC axons form synapses with neurons in the visual centers of the brain. Degeneration of these brain neurons has been reported after injury to the retina [71, 72]. In the mouse, 90–95 % of axons in the optic nerve cross the optic chiasm and progress toward the contralateral side of the brain [37]. Among them, approximately 70 % of axon terminals are localized in the superficial layer of the SC [38]. Damage resulting from retinal ischemia leads to defects in anterograde transport and loss of innervation to the SC [73], causing a significantly decreased visual evoked potential response [74, 75]. Topical treatment with the α2-adrenergic agonist brimonidine has been shown to maintain innervation of the SC and protect against loss of neuronal volume up to 3 months following transient ligation of the ophthalmic vessels in rats [73]. In our study, we describe significant decreases in areas of myelination as well as loss of pre- and post-synaptic proteins only in the contralateral hemisphere of the SC. Several studies have shown the neuroprotective action of JNK inhibitors, including SP600125 in the hippocampus [69, 70]. However, we believe this to be the first report of protection in the SC, or any visual center in the brain, provided by JNK inhibition. Long-term administration of SP600125 efficaciously preserved myelination in the superficial SC as well as maintained expression of an RGC pre-synaptic marker. In addition, JNK inhibition may promote subsequent preservation of structural integrity of post-synapses, which is necessary for active neurotransmission and neurotrophic factor exchange. The same trend was observed with only 7 days of dosing.

## Conclusions

In summary, we have shown JNK inhibitors protected RGCs against glutamate and TFW-induced cytotoxicity in primary cultured retinal neurons. Further, intraperitoneal injection of the JNK inhibitor SP600125 ameliorated retinal I/R-induced degeneration and functional loss of the inner neural retina and synaptic loss at RGC axon terminals in the SC. Our findings implicate that JNK activation, within hours immediately following reperfusion, plays a pivotal role in cell death and pathological progression in the retina and SC. This may be the critical therapeutic window for JNK inhibitors, at least in this acute model of retinal I/R injury. Together with previously published results, we suggest that the JNK signaling pathway is a prominent and critical path for neurodegeneration. Inhibition of this pathway represents a promising novel therapeutic target for preventing neurodegeneration in the visual axis related to retinal ischemic injury.

## Methods

### Animal

Female Sprague–Dawley rats (2–4 months of age) from Charles River Laboratories (Wilmington, MA) were used for retinal cell culture. Female C57BL/6J mice (10–12 weeks of age) from the Jackson Laboratory (Bar Harbor, ME) were used for transient retinal I/R studies. Animals were maintained in 12-h:12-h/light:dark cycle. All studies and animal care were performed as approved by the Institutional Animal Care and Use Committee at the University of North Texas Health Science Center and followed the Association for Research in Vision and Ophthalmology (ARVO) Statement for the Use of Animals in Ophthalmic and Vision Research.

### Mixed retinal cell culture

Culture of retinal neurons was performed as previously reported [30]. Briefly, rats were euthanized by CO_2_ asphyxiation and their eyes enucleated. The retina from each eye was dissected and incubated in a papain solution, containing 2 mg/mL papain (Sigma, St. Louis, MO), 0.4 mg/mL DL-cysteine (Sigma), and 0.4 mg/mL bovine serum albumin (Sigma) in Neurobasal medium (Gibco/Invitrogen, Carlsbad, CA), for 25 min at 37 °C, then washed 3 times with RGC culture medium (Neurobasal/B27 medium containing 100 units/mL penicillin (Sigma), 100 μg/mL streptomycin (Sigma), 1 mM pyruvate (Gibco/Invitrogen), 2 mM glutamine (Gibco/Invitrogen), 5 μg/mL insulin (Sigma), 100 μg/mL transferrin (Sigma), 100 μg/mL bovine serum albumin (Sigma), 60 ng/mL progesterone (Sigma), 16 μg/mL putrescine (Sigma), 40 ng/mL sodium selenite (Sigma), 40 ng/mL thyroxine (Sigma), 40 ng/mL tri-iodothyronine (Sigma), 50 ng/mL brain-derived neurotrophic factor (BDNF; Biosource, Camarillo, CA), 10 ng/mL ciliary neurotrophic factor (CNTF; Biosource), 10 ng/mL basic fibroblast growth factor (bFGF; Biosource), 5 μM forskolin (Sigma), and 1 % fetal calf serum (Atlas Biologicals, Fort Collins, CO)). Retinal pieces were triturated by passing through a fire-polished disposable glass pipette until cells were dispersed. Cell density in the suspension was assessed with a Coulter counter (Beckman Coulter, Fullerton, CA). 1 × 10^6^ to 3 × 10^6^ cells/well were placed onto poly-D-lysine- and laminin-coated 8-well chambered culture slides (surface area = 0.69 cm^2^/well; Becton Dickinson, Franklin Lakes, NJ) and cultured in 95 % air/5 % CO_2_ at 37 °C. These cell cultures contained RGC-enriched retinal neurons [30]. More than 90 % of the cells were neurons, which were positively labeled with neuron-specific enolase antibody. Approximately 20–30 % of these cells expressed RGC markers, Thy-1 and neurofilament-L. Morphologically, the Thy-1-positive cells had the characteristic appearance of neurons. After 2–3 days in culture, neurite outgrowth typically had 2–4 main branches of approximately 20–50 μm in length. Cells in culture were negative for arrestin (photoreceptor), glial fibrillary acidic protein (astroglia and Müller cell), glutamine synthetase (Müller cell), or ED1 (microglia). Less than 10 % of the cells were labeled with the protein kinase Cα antibody (rod bipolar cells) [30]. Characterization of this cell culture system is shown in Additional file 1: Table S1 and Figures S1–S8. For glutamate-induced toxicity studies, cells were pre-treated with vehicle or the indicated JNK inhibitors, SP600125 (Sigma) or TAT-JNK-III, a cell-permeable peptide inhibitor specific for JNK [76] (EMD Chemicals, Billerica, MA), for 30 min, followed by 100 μM glutamate (Sigma) for three days. For TNFα-induced toxicity studies, cells were pre-treated with vehicle or the indicated compounds for 30 min, followed by 10 ng/mL TNFα (Sigma) for three days. For trophic factor withdrawal (TFW) studies, three trophic factors, bFGF, BDNF, and CNTF, were removed from the culture medium. Cells were cultured in this medium with the indicated compounds for three days. At the end of the incubation period, the cells were fixed and labeled for Thy-1, a RGC marker, by immunocytochemistry (primary antibody against Thy-1, diluted 1:500; Chemicon, Temecula, CA; secondary antibody: Alexa fluor 594-labeled goat anti-mouse IgG, 1:300; Invitrogen/Molecular Probes, Carlsbad, CA). Cell survival was quantified by counting the Thy-1-positive cells in each well.

### Retinal I/R and SP600125 administration

Retinal I/R was induced as described previously [27]. Briefly, mice were anesthetized with a ketamine/xylazine/acepromazine cocktail (100/10/3 mg/kg) followed by cannulation of the anterior chamber with a 30 gauge needle connected to a reservoir containing sterile PBS. The reservoir was elevated to generate an intraocular pressure of 120 mmHg for 1 h to induce retinal ischemia. The JNK inhibitor SP600125 (5, 15, 30 mg/kg, 100 μL) or vehicle control (6 % DMSO in PBS, 100 μL) was intraperitoneally administered 2 h prior to retinal I/R, followed by once daily injection after I/R.

### Western blotting of retinal proteins

Whole retina was gently removed at indicated time points after I/R injury and homogenized in mammalian protein extraction reagent (MPER, Thermo Fisher Scientific Inc, Rockford, IL). Total protein was quantified using DC™ Protein Assay (Bio-RAD, Life Science Research, Hercules, CA). Total retinal protein (50 μg) in Laemmli buffer (Bio-RAD) and 0.5 % β-mercaptoethanol was electrophoresed in 10 % SDS-PAGE gels. Gels were transferred to Immobilon-P PVDF transfer membrane (EMD Millipore, Billerica, MA) followed by blocking in 10 % non-fat milk. Membranes were incubated overnight in 4 °C with primary antibodies for pJNK (#9251), total JNK (#9252), phospho-c-Jun (pc-Jun) (#2631), total c-Jun (#9165), and GAPDH (#2118) from Cell Signaling Technology (Danvers, MA), followed by incubation with secondary antibody conjugated with horse radish peroxidase (Cell Signaling Technology) for 1 h at room temperature. Protein expression was detected using SuperSignal West Femto Chemiluminescent substrate (Thermo Fisher Scientific, Rockford, IL) and Alpha Innotech Gel Documentation System with Flourchem 8900 software (ProteinSimple, Alpha Innotech, Santa Clara, CA). Immunoblots were quantified using the ImageJ program (National Institutes of Health, Bethesda, MD) and expression was normalized to GAPDH.

### Immunohistochemistry of retina

Ischemic or contralateral control eyes were gently removed from euthanized mice and placed in 4 % para-formaldehyde/PBS for 2 h followed by 20 % sucrose embedding. Eyes were placed in Tissue-Tek O.C.T compound (Sakura Finetek USA, Torrance, CA) and stored at −80 °C. Eyes were frozen-sectioned at 10 μm thickness and placed on Superfrost Plus Microscope Slides (Fisher Scientific, Hampton, NH). Slides were washed twice in PBS, blocked and permeabilized for 1 h using blocking buffer (PBS with 10 % horse serum and 0.1 % Triton X-100), then incubated with antibodies against pJNK (#4668), pc-Jun (#3270) (Cell Signaling Technology, Danvers, MA), and/or Tublin βIII (Tuj1 #801201, Biolegend, San Diego, CA), or PKCα (ab11723), Syntaxin (ab112198) (Abcam plc. Cambridge, MA) for 16 h at 4 °C, followed by treatment of secondary antibody conjugated with Alexaflour 488 and Alexaflour 568 (Invitrogen) for 1 h at room temperature. Slides were mounted with cover slips using ProlongGold anti-fade reagent with DAPI (Molecular Probes, Life Technologies, Grand Island, NY). All retinal images were taken using Zeiss LSM Meta Confocal microscope system (located in Johns Hopkins University, School of Medicine, Baltimore, MD) and Zen 2009 software (ZEISS, Thornwood, NY).

### Histology of retina

Retinal layer thickness and cell numbers in ganglion cell layer (GCL) were assessed as previously described [27]. Briefly, thicknesses of individual layers or whole retina (from nerve fiber layer (NFL) to outer nuclear layer (ONL), excluding outer segments) were measured using the ImageJ program (NIH) at every quarter point of each entire retinal cross-section (from ora serrata to ora serrata) and averaged. Four retinal cross-sections were used per eye and their results averaged. Cell numbers in GCL were counted from each of the four cross-sections from each eye and averaged.

### Spectral domain optical coherence tomography (SD-OCT)

The SD-OCT ophthalmic Imaging System (Bioptigen, Durham, NC) was used for retinal scanning. Mice were anesthetized using a ketamine/xylazine cocktail (100/10 mg/kg) and placed on a mouse holder. Using InVivoVue software (Bioptigen), each retina was scanned with rectangular scanning mode (1.2 × 1.2 mm) consisting of 1000 A scans per B scan × 100 B scans. Images from superior, central, and inferior regions were analyzed using ImageJ software with four measurements at quarter points for thicknesses of the whole retinal layer, inner plexiform layer (IPL), inner nuclear layer (INL), and ONL.

### Scotopic electroretinography (ERG)

After 16 h of dark adaptation, mice were anesthetized with isoflurane and connected to the HMsERG system (Ocuscience, Rolla, MO) with body temperature controlled at 37 °C. The study eye was exposed to a series of light flashes of various intensities (0.1, 0.3, 1, 3, 10, and 25 cd/m^2^). Amplitudes and implicit times of ERG waveforms were measured and analyzed.

### Brain histology and immunohistochemistry

For black gold staining, blocks of mice brains bracketing the SC (3 mm to 6 mm caudal to bregma) were frozen-sectioned at 40 μm thickness and placed on slides. Slides were incubated with 0.3 % Black Gold II solution (Histo-Chem, Jefferson, AR) at 60 °C as instructed by the manufacturer. Before mounting, slides were washed in PBS and dehydrated in a series of 95 % ethanol (2 min), 100 % ethanol (2 min) and xylene (2 min). All bright-field images were taken using Nikon eclipse Ti inverted microscope and CRi Nuance FX multispectral imaging system with Nuance 3.0 software. Cross-sectional areas of SC of all slides were measured from the myelinated SC layer III to superficial layer in each hemisphere as previously described [77] using the ImageJ polygonal selection method. The cross-sectional areas of SC of all slides from each hemisphere of the same brain were averaged and reported.

For immunohistochemistry, brains were frozen-sectioned at 10 μm thickness. Prior to applying primary antibody, slides were hydrated in PBS for 10 min and blocked with 10 % goat serum containing 0.1 % Triton X-100 in PBS. Anti-VGLUT2 (ab79157, Abcam) and anti-PSD95 (ab18258, Abcam) antibodies were applied at 4 °C overnight. Slides were washed and incubated with secondary antibodies conjugated with Alexaflour 488 or Alexaflour 568 for 1 h at room temperature. All fluorescence images were taken using the same system described above. Images were taken using Nikon eclipse Ti inverted microscope (Nikon) and CRi Nuance FX multispectral imaging system (Caliper Life Sciences, Hopkinton, MA). All background autofluorescence was subtracted using Nuance 3.0 software (Caliper Life Sciences).

### Statistics

Statistical analysis was performed using GraphPad Prism Version 5.0 (GraphPad Software, San Diego, CA). Student’s *t*-test was used to compare two experimental groups. One-way or two-way ANOVA was used to compare among three or more groups. The specific statistical method used is described in the respective figure legends. All data are expressed as means ± standard error of mean (SEM). A *p* value less than 0.05 is considered statistically significant.

## Additional file

Additional file 1:
**Table S1.** Summary of distribution of cell markers in the mixed rat retinal cell culture. **Figure S1.** Representative images showing colocalization of Thy-1 and neurofilament-L (NF-L) by immunocytochemistry in cultured rat retinal cells*.* **Figure S2.** Representative images showing lack of colocalization of Thy-1 and arrestin by immunocytochemistry in cultured rat retinal cells*.* There were no arrestin positive cells in culture. **Figure S3.** Representative images showing lack of colocalization of Thy-1 and ED-1 (CD68) by immunocytochemistry in cultured rat retinal cells*.* There were no ED-1 positive cells in culture. **Figure S4.** Representative images showing lack of colocalization of Thy-1 and glutamine synthetase (GS) by immunocytochemistry in cultured rat retinal cells*.* There were no GS positive cells in culture. **Figure S5.** Representative images showing lack of colocalization of Thy-1 and GFAP by immunocytochemistry in cultured rat retinal cells. There were no GFAP positive cells in culture. The lower right-hand panel includes brain astrocytes as a positive control for the anti-GFAP antibody. **Figure S6.** Representative images showing lack of colocalization of Thy-1 and PKCα by immunocytochemistry in cultured rat retinal cells*.* There were very few PKCα positive cells in culture. **Figure S7.** Representative images showing partial colocalization of Thy-1 and neuron-specific enolase (NSE) by immunocytochemistry in cultured rat retinal cells. **Figure S8.** Effect of glutamate on RGC morphology. Cells were treated with glutamate (100 μM) or vehicle for 3 days. (Top panels) The cells were labeled with anti-Thy-1 antibody (red) and DAPI nuclear stain (blue). (Bottom panels) The corresponding phase-contrast image. **Figure S9.** Retinal c-Jun phosphorylated c-Jun was attenuated by SP600125 administration in 24 h after I/R injury. Phosphorylated c-Jun was detected (green fluorescence). Tuj-1 immunofluorescence (red) was used as RGC marker. DAPI staining (blue) represents cell nuclei for counter staining. (A) Vehicle, (B) SP600125. (PDF 362 kb)

## Abbreviations

ANOVA: analysis of variance
ASK1: apoptosis signal regulating kinase 1
ATF-2: activating transcription factor 2
BDNF: brain-derived neurotrophic factor
bFGF: basic fibroblast growth factor
CNTF: ciliary neurotrphic factor
DAPI: 4′ 6-diamido-2-phenylindole
ELK-1: ETS domain containing protein
ERG: electroretinography
GAPDH: glyceraldehyde 3-phosphate dehydrogenase
GCL: ganglion cell layer
GFAP: glial fibrillar acidic protein
Glu: glutamate
I/R: ischemia/reperfusion
INL: inner nuclear layer
IOP: intraocular pressure
IPL: inner plexiform layer
JNK: cJun N-terminal kinase
MAP3K: mitogen activated protein kinase kinase kinase
MLK: mixed lineage kinase
MPER: mammalian protein extraction reagent
MPTP: 1-methyl-4-phenol-1,2,3,6-tetrahydropyridine
NF-L: neurofilament light
NIH: National Institutes of Health
NMDA: N-methyl-D-aspartate
NSE: neuron specific enolase
OCT: optimal cutting temperature compound
OPL: outer plexiform layer
pc-Jun: phopho-c-Jun
PD: Parkinson’s Disease
pJNK: phospho-c-Jun N-terminal kinase
PKCα: protein kinase C alpha
PSD95: postsynaptic density protein 95
PVDF: polyvinylidine fluoride
RGC: retinal ganglion cell
ROS: reactive oxygen species
SC: superior colliculus
SD-OCT: spectral domain-optical coherence tomography
SEM: standard error of the mean
Stat3: Signal transducer and activator of transcription 3
TFW: trophic factor withdrawal
TNFα: tumor necrosis factor alpha
Tuj-1: neuron specific class III β-tubulin
VGTUT2: vesicular glutamate transporter 2
WRL: whole retinal layer
