# Supplementary material for: In vitro and in vivo neuroprotective effects of cJun N-terminal kinase inhibitors on retinal ganglion cells
Source: Mol Neurodegener. 2016 Apr 21;11:30. doi: 10.1186/s13024-016-0093-4 (PMC4839164; doi:10.1186/s13024-016-0093-4)
Supplement: Additional file 1: — Table S1. Summary of distribution of cell markers in the mixed rat retinal cell culture. Figure S1. Representative images showing colocalization of Thy-1 and neurofilament-L (NF-L) by immunocytochemistry in cultured rat retinal cells. Figure S2. Representative images showing lack of colocalization of Thy-1 and arrestin by immunocytochemistry in cultured rat retinal cells. There were no arrestin positive cells in culture. Figure S3. Representative images showing lack of colocalization of Thy-1 and ED-1 (CD68) by immunocytochemistry in cultured rat retinal cells. There were no ED-1 positive cells in culture. Figure S4. Representative images showing lack of colocalization of Thy-1 and glutamine synthetase (GS) by immunocytochemistry in cultured rat retinal cells. There were no GS positive cells in culture. Figure S5. Representative images showing lack of colocalization of Thy-1 and GFAP by immunocytochemistry in cultured rat retinal cells. There were no GFAP positive cells in culture. The lower right-hand panel includes brain astrocytes as a positive control for the anti-GFAP antibody. Figure S6. Representative images showing lack of colocalization of Thy-1 and PKCα by immunocytochemistry in cultured rat retinal cells. There were very few PKCα positive cells in culture. Figure S7. Representative images showing partial colocalization of Thy-1 and neuron-specific enolase (NSE) by immunocytochemistry in cultured rat retinal cells. Figure S8. Effect of glutamate on RGC morphology. Cells were treated with glutamate (100 μM) or vehicle for 3 days. (Top panels) The cells were labeled with anti-Thy-1 antibody (red) and DAPI nuclear stain (blue). (Bottom panels) The corresponding phase-contrast image. Figure S9. Retinal c-Jun phosphorylated c-Jun was attenuated by SP600125 administration in 24 h after I/R injury. Phosphorylated c-Jun was detected (green fluorescence). Tuj-1 immunofluorescence (red) was used as RGC marker. DAPI staining (blue) represents cell nuclei for counter [file 13024_2016_93_MOESM1_ESM.pdf]

Table S1: Summary of distribution of cell markers in the mixed rat retinal cell culture

Marker	Cells Labeled	% All Cells
Thy-1	RGC	≈ 30%
NF-L	RGC	≈ 30%<br>(co-localized with Thy-1)
Arrestin	Photoreceptor	0%
CD68 (ED-1)	Microglia	0%
Glutamine synthetase	Muller	0%
GFAP	Astroglia/Müller	0%
PKCα	Rod bipolar	≈ 5%
NF200	RGC + Horizontal	>95%
NSE	All neurons	>95%

Supplementary Figures

Figure S1

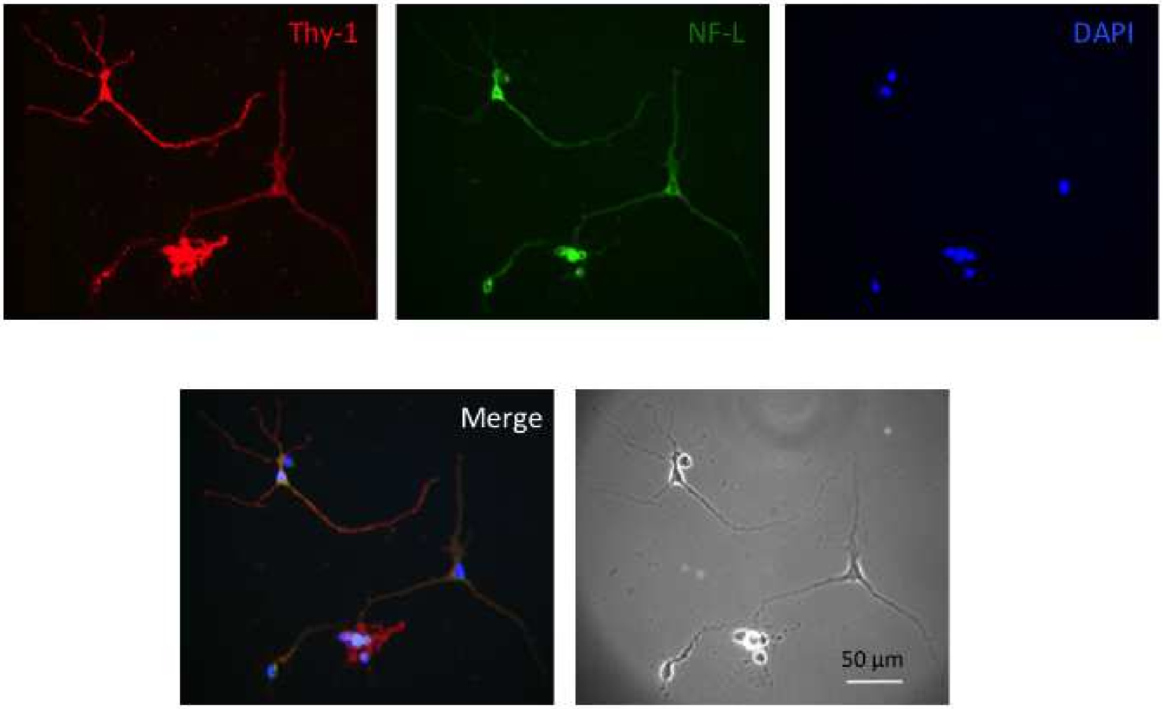

Figure S2

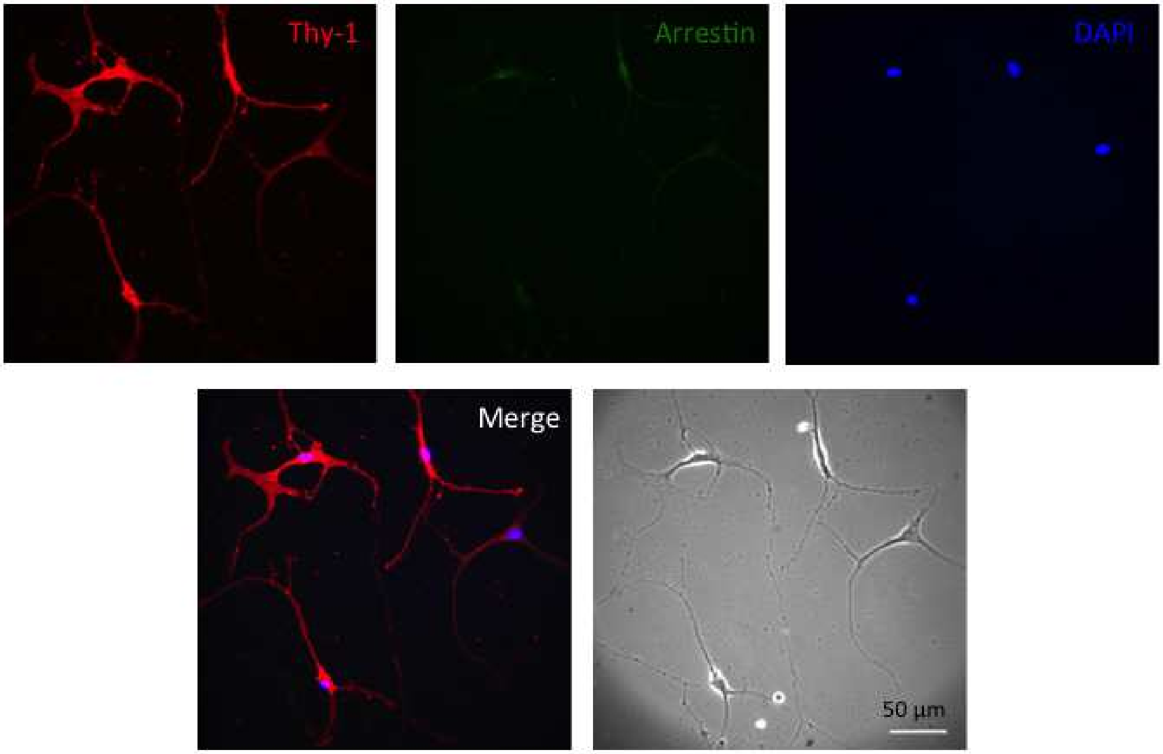

Figure S3

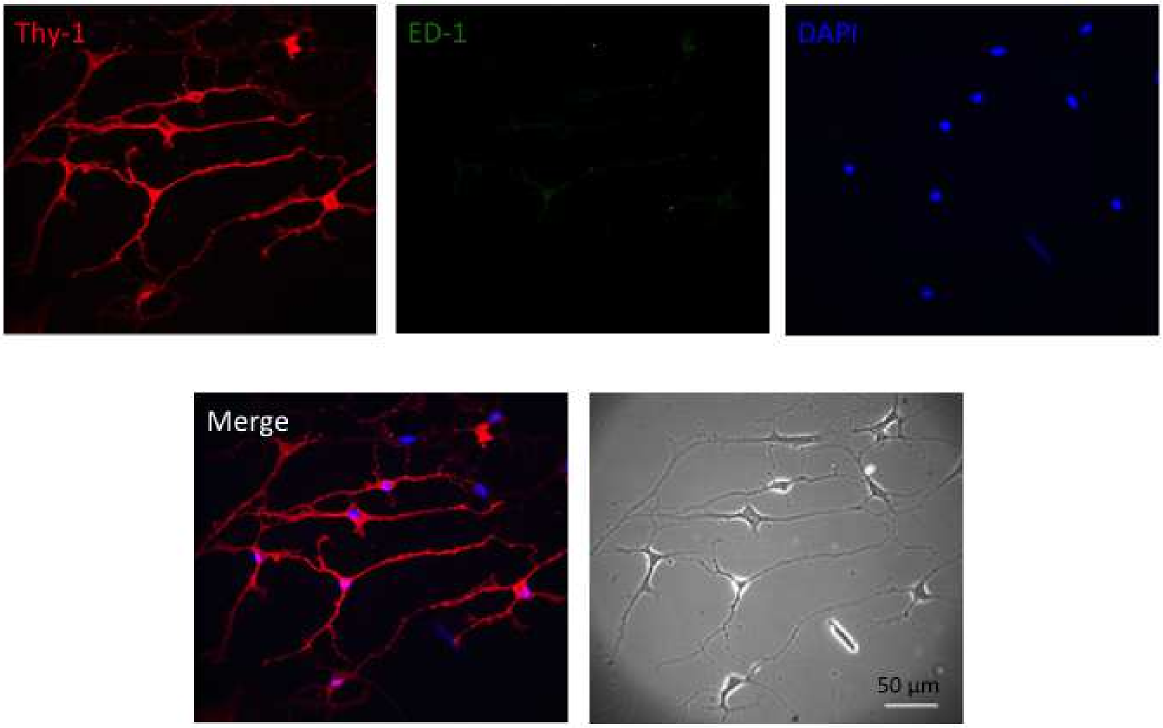

Figure S4

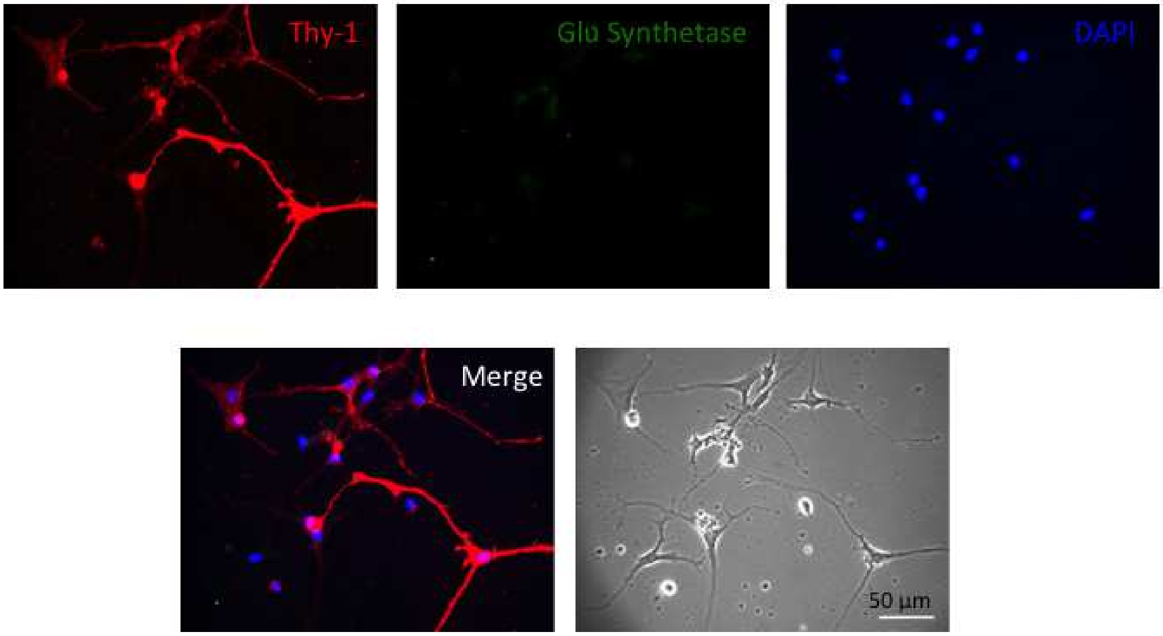

Figure S5

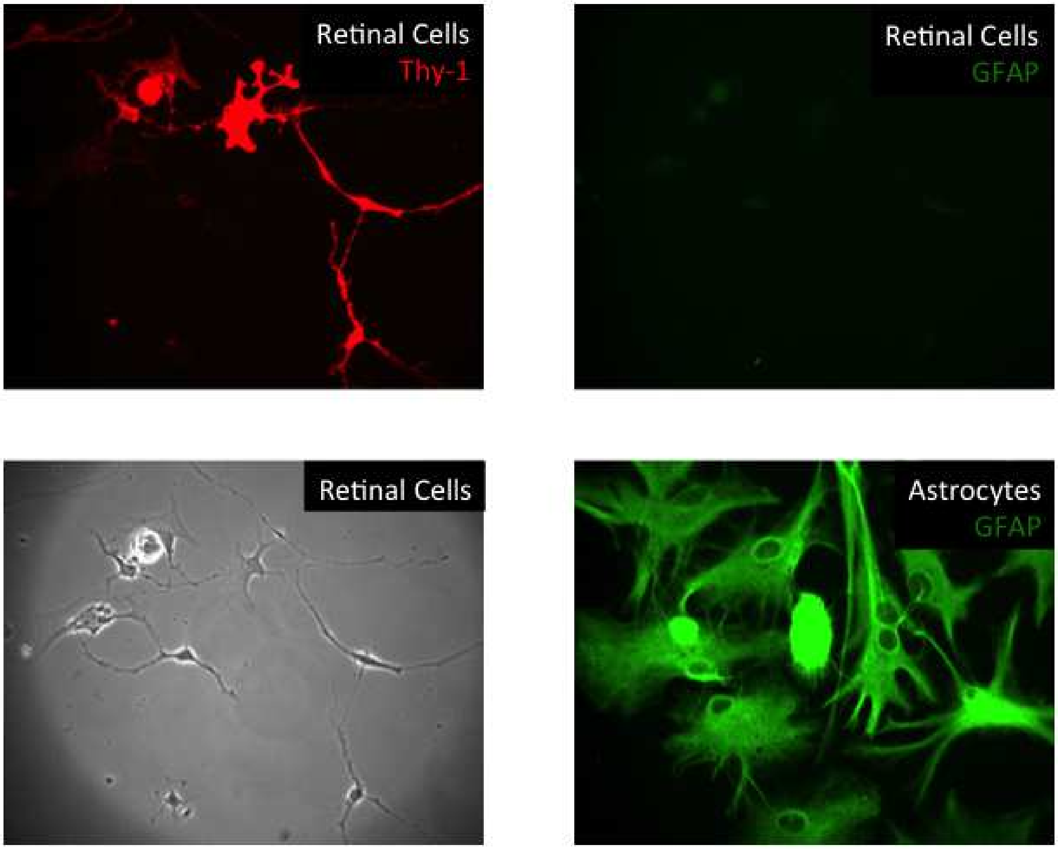
Retinal Cells
Thy-1
Retinal Cells
GFAP
Retinal Cells
Astrocytes
GFAP

Figure S6

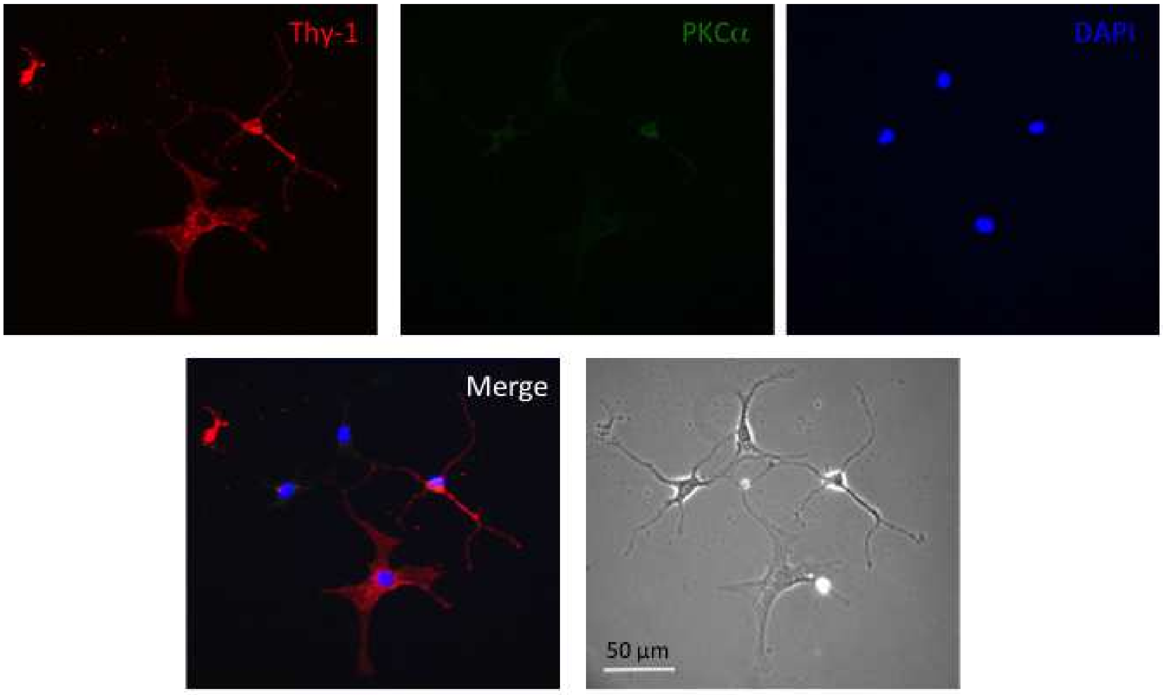

Figure S7

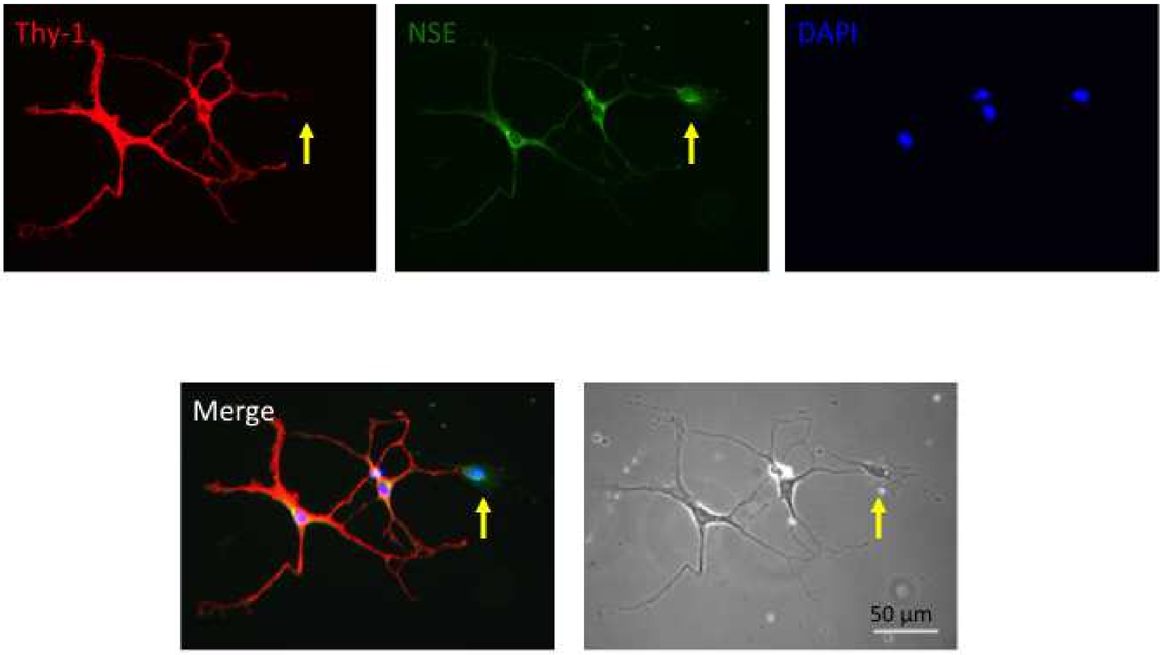

Figure S8

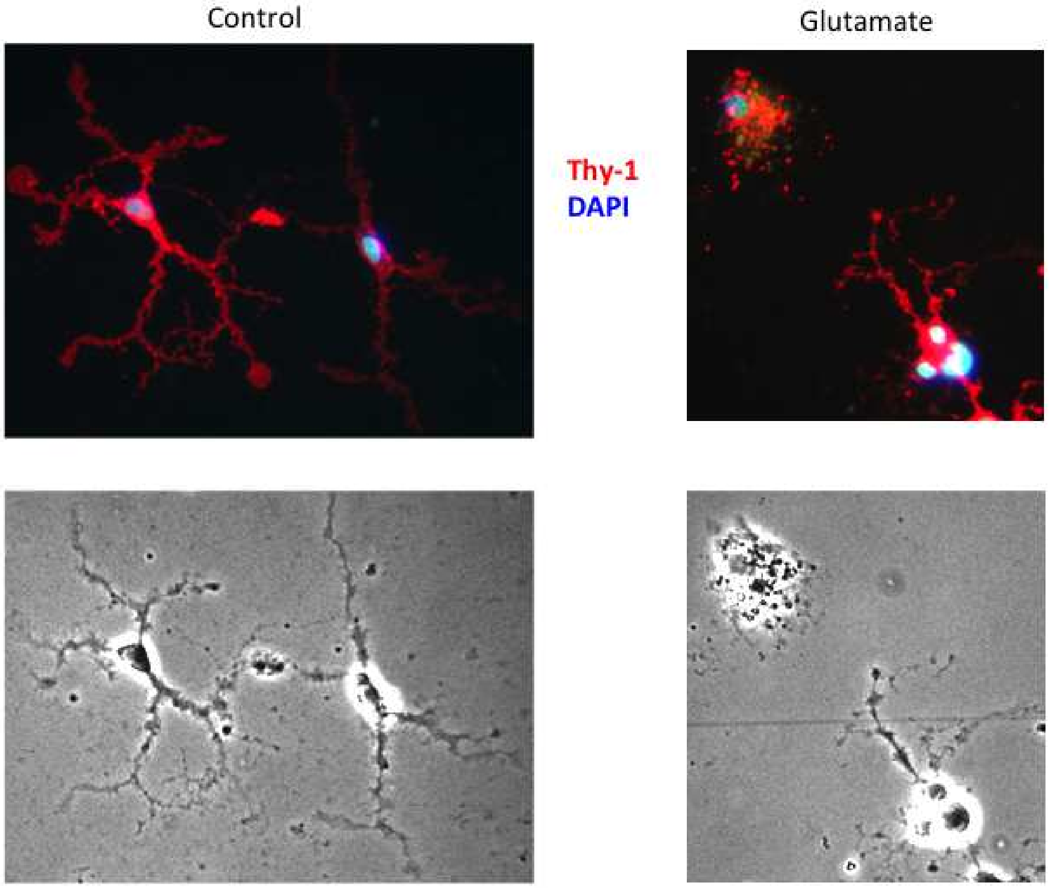

Figure S9

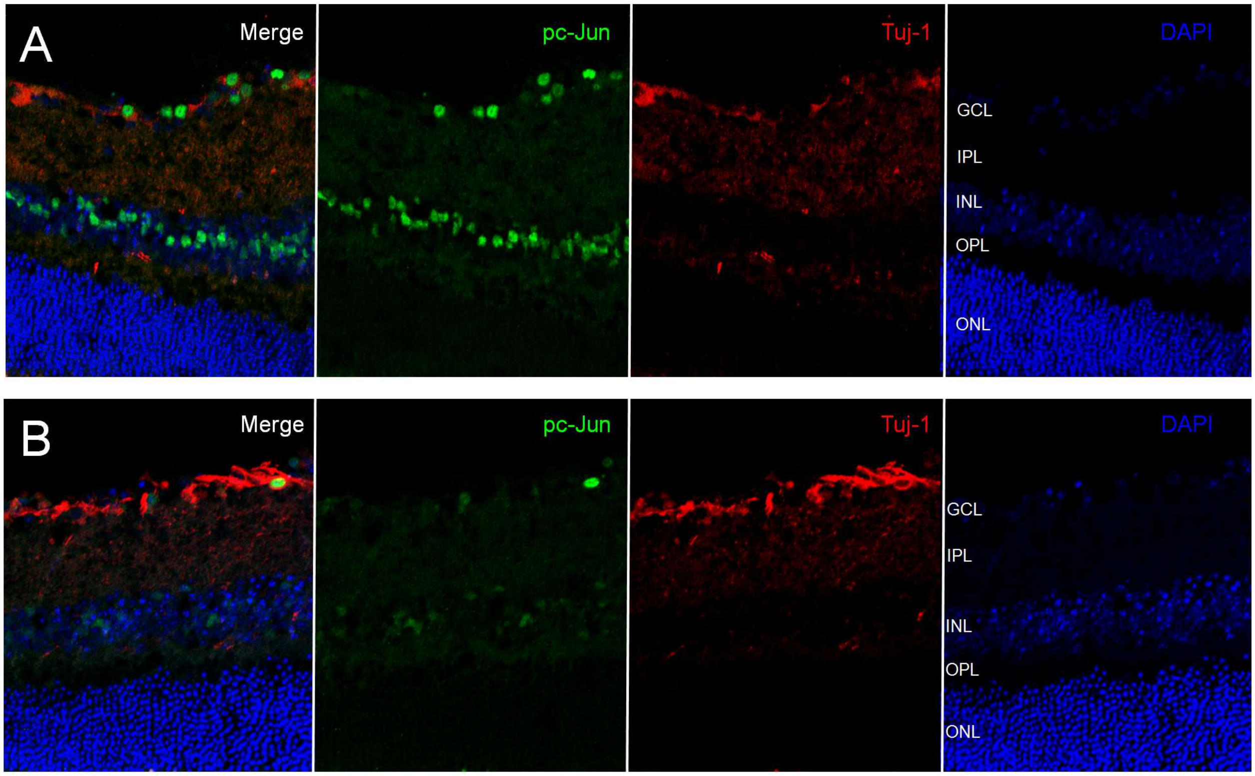
A
Merge
pc-Jun
Tuj-1
DAPI
GCL
IPL
INL
OPL
ONL
B
Merge
pc-Jun
Tuj-1
DAPI
GCL
IPL
INL
OPL
ONL
